# Evaluating Health Financing Typologies Through Healthy Life Expectancy and Infant Mortality: Evidence from OECD Countries and Türkiye

**DOI:** 10.3390/healthcare13233149

**Published:** 2025-12-02

**Authors:** Salim Yılmaz, Yusuf Çelik

**Affiliations:** Department of Health Management, Faculty of Health Sciences, Acibadem Mehmet Ali Aydinlar University, Istanbul 34752, Türkiye; yusuf.celik@acibadem.edu.tr

**Keywords:** health financing models, healthy life expectancy, infant mortality, OECD countries, Türkiye

## Abstract

**Highlights:**

**What are the main findings?**

- High-Public-Spending systems achieved an 81.2% success rate in top-quartile health performance, whereas Moderate/Emerging systems exhibited only 8.3% success, with Türkiye ranking 36th of the 38 countries (TOPSIS score = 24.8, 45% below cluster average).
- Sex-disaggregated analysis revealed that low-resource systems magnify gender disparities (HALE gap 3.43 vs. 1.66 years; male excess infant mortality 1.04 vs. 0.53 per 1000), with Türkiye exhibiting the third-highest male infant mortality gap globally.

**What are the implications of the main findings?**

- Robust public financing (>USD 3500 per capita, >7% GDP) is necessary and nearly sufficient for superior outcomes—81% of High-Public-Spending systems achieve top performance versus only 8% of low-resource systems, demonstrating that adequate financing is non-negotiable.
- Türkiye requires fundamental increases in public investment (at minimum, doubling from USD 813 to ~1900 per capita) alongside targeted neonatal care expansion to address extreme efficiency deficits and gender disparities in infant mortality.

**Abstract:**

**Background/Objectives**: The structure and adequacy of health financing critically shape population health outcomes. This study examines financing typologies in relation to healthy life expectancy (HALE) and infant mortality across 38 OECD countries and Türkiye (2000–2021), quantifying financing model effectiveness and sex-disaggregated disparities. **Methods:** Time-weighted averages (exponential weighting, λ = 1.5) emphasized recent policy environments while preserving historical context. Principal component analysis addressed multicollinearity among six financial indicators. Multidimensional scaling (stress = 1.16 × 10^−12^) and K-means clustering identified four financing typologies. TOPSIS composite scores measured proximity to ideal outcomes (maximum HALE, minimum infant mortality), with success rates calculated as the percentage achieving top-quartile performance (TOPSIS ≥ 70). Sex-disaggregated analysis examined gender gaps across clusters. **Results:** High-Public-Spending systems achieved an 81.2% success rate (mean TOPSIS = 76.0), those with Balanced High-Expenditure achieved 77.8%, whereas Moderate/Emerging systems exhibited only 8.3% success. Türkiye ranked 36th of the 38 (TOPSIS = 24.8), 45% below cluster average, with extreme deficits in HALE (percentile = 15.8%) and infant mortality (7.9%). Low-resource systems showed significantly wider gender gaps (HALE: 3.43 vs. 1.66 years; infant mortality male excess: 1.04 vs. 0.53 per 1000; *p* < 0.01), with Türkiye demonstrating the third-highest male excess mortality globally (1.69 per 1000), indicating critical neonatal care deficiencies. **Conclusions:** Robust public financing (>USD 3500 per capita, >7% GDP) is necessary and nearly sufficient for superior outcomes, with success rates differing 10-fold between high- and low-resource systems (81% vs. 8%). Türkiye’s extreme underperformance reflects both inadequate public investment (USD 813 per capita, 22% of high-performing averages) and efficiency deficits requiring doubled expenditure alongside targeted maternal–child health interventions.

## 1. Introduction

Health systems face increasing pressure to provide equitable, high-quality care while maintaining financial sustainability. The World Health Organization emphasizes that how a system is financed matters as much as how much is spent, since the financing model directly influences access, efficiency, and the distribution of health outcomes [1] Cross-national evidence consistently links higher shares of public spending to favorable population health indicators, such as longer healthy life expectancy and lower infant mortality [1,2,3]. Yet even countries allocating similar proportions of national income to health can experience markedly different outcomes. For instance, the expansion of publicly funded Medicaid coverage in the United States from 2010 to 2022 reduced all-cause mortality among low-income adults by approximately 20%, saving an estimated 27,400 lives by improving access to preventive services [4]. These findings highlight that who pays and how funds are allocated often matter more than total spending alone.

Türkiye provides a telling example. Since the launch of the 2003 Health Transformation Program, coverage has expanded and physical infrastructure has improved, culminating in the provision of nationwide General Health Insurance in 2008 [5]. These reforms enhanced financial risk protection but also increased private-sector participation and public–private partnerships, gradually shifting the system toward a hybrid model [6]. Despite public funds accounting for the majority of spending, per-capita outlays remain below the OECD average, preventive services receive a limited budget share, and regional and income-related disparities persist [7]. While rising public expenditure can reduce maternal and infant mortality and extend healthy life expectancy [8], Türkiye’s outcomes lag behind countries with similar public-financing ratios, revealing a disconnect between financing structure and effectiveness—which is particularly pronounced in vulnerable populations requiring resource-intensive maternal–child health interventions.

Two gaps in the literature motivate this study. First, most cross-country analyses treat health expenditure as a single aggregate, overlooking the distinct incentives of compulsory public contributions, voluntary insurance, and out-of-pocket payments [9]. Second, cross-sectional comparisons ignore temporal dynamics: countries with identical current spending ratios may have arrived via different policy trajectories (gradual public expansion vs. austerity-driven retrenchment), yet most studies use single-year snapshots that erase this history [10]. Because health outcomes reflect cumulative investments and institutional path dependencies rather than instantaneous spending levels, analyses relying solely on contemporaneous data cannot distinguish structurally different systems that temporarily converge in aggregate statistics. Addressing these gaps requires methods that (i) disaggregate spending components, (ii) consider their joint dynamics over time, and (iii) link resulting typologies to concrete health outcomes [11,12,13].

This study evaluates 38 OECD countries and Türkiye using longitudinal data from 2000 to 2021 by

- Converting 22-year spending trajectories into time-weighted cross-sectional indicators using exponential weighting (λ = 1.5) that emphasizes recent policy environments (2015–2021) while preserving historical context (2000–2014), capturing cumulative institutional development rather than arbitrary single-year snapshots;
- Addressing multicollinearity among correlated financial measures through principal component analysis, which reduces six spending indicators (per-capita and GDP-share measures for out-of-pocket, voluntary, and compulsory public spending) to three orthogonal components explaining 78–99% of within-group variance;
- Positioning countries within a three-dimensional health-financing space using multidimensional scaling ($stress=1.16\times 10^{-12}$) and identifying four coherent spending typologies through K-means clustering: High-Public-Spending (n = 16), Balanced High-Expenditure (n = 9), Moderate/Emerging (n = 12), and US Voluntary-Dominant (n = 1) systems;
- Quantifying financing model effectiveness through composite performance scores using the TOPSIS (Technique for Order of Preference by Similarity to Ideal Solution) method, which measures each country’s proximity to ideal health outcomes (maximum HALE, minimum infant mortality) on a 0–100 scale, enabling the calculation of success rates (percentage of cluster members achieving top-quartile performance, TOPSIS ≥ 70) that systematically compare resource mobilization strategies;
- Examining sex-disaggregated outcomes to assess whether financing structures differentially affect gender disparities in HALE and infant mortality, revealing whether inadequate public investment magnifies biological vulnerabilities through constrained access to maternal–child health services and neonatal intensive care.

By situating Türkiye in this international context, the study evaluates whether its Moderate/Emerging financing structure—characterized by low absolute public investment (USD 813 per capita, 3.50% GDP) despite formal universal coverage—performs in line with peer countries and examines why countries with nominally similar public financing shares produce substantially different health outcomes. Using TOPSIS performance scores, the analysis demonstrates that Türkiye ranks 36th of the 38 countries (score = 24.8), 45% below its financing cluster average and exceeded in poor performance only by Colombia and Mexico, indicating that outcome deficits reflect not merely moderate resources but extreme efficiency failures in translating nominal coverage into equitable health improvements.

## 2. Materials and Methods

This secondary data analysis using longitudinally derived cross-sectional indicators investigates the relationship between health-financing structures and health outcomes—specifically, healthy life expectancy at birth (HALE) and infant mortality—across 38 OECD countries and Türkiye using longitudinal data from 2000 to 2021. Data were sourced from official OECD Health Statistics and WHO Global Health Observatory platforms [3,14]. Six spending indicators were analyzed: out-of-pocket expenditure (USD per capita and % GDP), voluntary/private insurance expenditure (USD per capita and % GDP), and compulsory public expenditure (USD per capita and % GDP). HALE data, available from 2000 onward, determined the temporal scope; this 22-year window encompasses major health system reforms including post-2008 financial crisis adjustments [15], Affordable Care Act implementation (USA), and universal health coverage expansions in emerging economies.

Annual data (22 observations per country, 2000–2021) were summarized into single cross-sectional values using an exponential time-weighted average, which emphasizes recent trends while preserving historical context [16,17]. The weighting formula applied was

$${\bar{x}}_{i}=\frac{\sum_{t=1}^{T}x_{it}\times e^{\lambda *[(t-t_{0})/(T-t_{0})]}}{\sum_{t=1}^{T}e^{\lambda *[(t-t_{0})/(T-t_{0})]}}$$

Here, e is Euler’s number; t is the year of the t-th observation; $t_{0}$ is the earliest year in the series (normalized to zero); λ controls the slope of temporal weighting; and T denotes the total number of available years. The choice of λ=1.5 was determined through multi-criteria optimization, balancing clustering quality (silhouette coefficient) and predictive validity (variance explained in outcome variables). This value yields a weight ratio of approximately 4.5:1 between the most recent and earliest observations, providing moderate emphasis on contemporary policy environments while maintaining statistical stability [16]. As an extension of the primary indicators, sex-specific HALE and infant mortality rates for all OECD countries and Türkiye (2000–2021) were processed using the same exponential weighting scheme, allowing male and female trajectories to be incorporated consistently into the temporal model. Gender gaps were computed as

$$HALE_{gap}=HALE_{female}-HALE_{male}$$

$$InfantMortality_{gap}=InfantMortality_{male}-InfantMortality_{female}\;$$

with positive values indicating female longevity advantage and male excess mortality, respectively. One-way ANOVA tested whether gender gaps differed significantly across the four financing clusters (High-Public-Spending, Balanced High-Expenditure, Moderate/Emerging, US Voluntary-Dominant). Statistical significance was assessed at α = 0.05 with Tukey’s HSD post-hoc tests for pairwise comparisons. Sensitivity analyses with λ∈{1.0, 1.5, 2.0, 2.5, 3.0} confirmed the robustness of cluster assignments and outcome associations (see Supplementary Material for details).

To reduce multicollinearity, principal component analysis (PCA) was applied separately to each spending group, retaining the first principal component (PC1) for each: Group 1: out-of-pocket spending and its GDP share (PC1 variance explained: 77.87%); Group 2: voluntary spending measures (98.69%); and Group 3: compulsory public spending measures (93.53%). Overall, the first principal components captured 78–99% of within-group variance, effectively consolidating correlated spending indicators into orthogonal dimensions while eliminating multicollinearity (all loadings |r|>0.88).

Multidimensional scaling (MDS) was applied to visualize country positions in the three-dimensional principal component space and assess distance preservation. MDS is a dimensionality reduction technique that constructs a low-dimensional configuration of observations while optimally preserving pairwise dissimilarities from the original high-dimensional space. Using the classical metric MDS algorithm, we computed Euclidean distances between all country pairs based on their PC1 scores from the three financing groups (out-of-pocket, voluntary, and compulsory public spending), yielding a 38 × 38 distance matrix. The algorithm iteratively positioned countries in k-dimensional space to minimize stress-1, defined as [18]

$$Stress\text{-}1=\sqrt{\frac{\sum_{i<j}{\left(d_{ij}-{\hat{d}}_{ij}\right)}^{2}}{\sum_{i<j}d_{ij}^{2}}}$$

We evaluated MDS solutions from one to five dimensions. The three-dimensional solution achieved near-perfect fit (stress-1 = 1.16 × 10^−12^), indicating complete preservation of the original distance structure without meaningful distortion. Goodness-of-fit was further assessed via a Shepard diagram (Supplementary Figure S1), which plots original distances against MDS-derived distances; the near-perfect linear alignment (Pearson r = 1.000) confirmed that the three-dimensional representation faithfully captured all pairwise country relationships. This three-dimensional MDS space was retained for subsequent clustering analysis.

The optimal number of clusters was determined through combined evaluation of the Elbow method (plotting within-cluster sum of squares against k) and average silhouette width (measuring cluster cohesion and separation). The Elbow method showed diminishing returns after k = 4, while silhouette analysis indicated adequate cluster quality at k = 4 (silhouette = 0.318), balancing parsimony with substantive interpretability. K-means clustering was applied to the three-dimensional MDS coordinates using Euclidean distance, with 50 random initializations to ensure global optimization. This procedure assigned countries to four distinct health-financing typologies: High-Public-Spending (n = 16), Balanced High-Expenditure (n = 9), Moderate/Emerging (n = 12), and US Voluntary-Dominant (n = 1) systems.

The K-means algorithm was initialized with 50 random starts (nstart = 50) and 100 maximum iterations (iter.max = 100) to ensure convergence to the global optimum. Cluster validity was assessed through: (i) ratio of between-cluster variance to total variance (0.512, indicating 51.2% variance explained), (ii) average silhouette width (0.318), and (iii) substantive coherence verified by examining cluster characteristics and outcome associations. Cluster stability was confirmed via cross-validation with alternative random seeds, yielding 100% classification agreement across initializations (detailed in Supplementary Material).

To contextualize Türkiye’s financing structure, Euclidean distances from Türkiye to all other countries were calculated in the MDS space and ranked. These distance rankings (Table 1), interpreted alongside cluster assignments and financing characteristics, reveal that Türkiye’s nearest neighbors (Colombia, Poland, Slovak Republic, and Estonia) share its Moderate/Emerging cluster membership, while high-performing Western European systems (Denmark, France, and Germany) are positioned at substantially greater distances (>4.0 units), underscoring structural financing differences beyond aggregate spending ratios.

A second clustering analysis was conducted using two outcome variables—healthy life expectancy at birth (HALE) and infant mortality rate—to develop a performance-based classification of countries. K-means clustering (k = 3) applied to standardized outcome data identified three distinct performance tiers: (i) High HALE and Low Infant Mortality (best-performing countries, n = 27: predominantly Western European and high-income systems achieving HALE > 44 years and infant mortality < 4 per 1000 live births); (ii) Moderate Performance (intermediate outcomes, n = 8: Central/Eastern European countries plus the United States, with HALE 41–43 years and infant mortality 4–7); and (iii) Low HALE and High Infant Mortality (worst-performing countries, n = 3: Türkiye, Mexico, and Colombia, with HALE < 41 years and infant mortality > 7). These outcome clusters were then cross-tabulated with the four financing typologies derived from MDS and K-means analysis: High-Public-Spending (n = 16), Balanced High-Expenditure (n = 9), Moderate/Emerging (n = 12), and US Voluntary-Dominant (n = 1) systems.

To quantify the effectiveness of different financing models in achieving favorable health outcomes, we computed a composite performance score using the TOPSIS (Technique for Order of Preference by Similarity to Ideal Solution) method [19]. HALE and infant mortality values were normalized to [0,1] scales (higher values indicating better performance), and each country’s distances from the ideal point (HALE = max., infant mortality = min.) and anti-ideal point (HALE = min., infant mortality = max.) were calculated. The TOPSIS score represents the relative closeness to the ideal solution, ranging from 0 (worst) to 100 (best). Success was defined as TOPSIS scores ≥ 70, empirically determined as the 75th percentile threshold across all countries, representing top-quartile performance.

All analyses were performed using R version 4.4.2. The following packages were employed: FactoMineR v2.8 for principal component analysis [20], MASS v7.3-60 for classical multidimensional scaling [21], cluster v2.1.4 for K-means clustering and silhouette validation [22], factoextra v1.0.7 for cluster visualization [23], ggplot2 v3.4.2 for publication-quality graphics [24], ggrepel v0.9.3 for non-overlapping text labels, gridExtra v2.3 for multi-panel figure arrangement, and dplyr v1.1.2 and tidyr v1.3.0 for data manipulation. Package selection prioritized computational efficiency (optimized PCA algorithms), methodological rigor (classical MDS with established stress metrics), reproducibility (deterministic K-means with fixed random seeds), and publication-quality output.

## 3. Results

The analysis first identifies and maps health-financing structures to reveal spatial patterns. These typologies are then compared with performance clusters based on HALE and infant mortality to evaluate their alignment with health outcomes (outcome-based clustering).

### 3.1. Health-Spending Typologies and Spatial Patterns

This subsection delineates the main clusters of health-financing models among OECD countries and Türkiye using time-weighted indicators and multidimensional scaling (MDS). Four distinct typologies emerged: High-Public-Spending Systems, Balanced High-Expenditure Systems, Moderate/Emerging Systems, and the US Voluntary-Dominant Model, capturing structural differences in how countries finance health systems. Türkiye is classified within the Moderate/Emerging Systems cluster, positioned closest to Central and Eastern European countries (Colombia, Poland, Slovak Republic, and Estonia), indicating a hybrid financing structure characterized by moderate compulsory spending coupled with substantial out-of-pocket contributions. To assess whether these financing structures translate into measurable health benefits, the subsequent sections examine cross-country outcomes in terms of HALE and infant mortality.

Annual data from 38 OECD countries and Türkiye (2000–2021) were converted into time-weighted averages using exponential weighting (λ = 1.5) to emphasize recent policy developments while retaining historical context. Principal component analysis (PCA) reduced multicollinearity among correlated financial indicators. For each spending group—out-of-pocket, voluntary, and compulsory public expenditure—the first principal component (PC1) was retained, explaining 77.87%, 98.69%, and 93.53% of within-group variance, respectively. The high variance explained by PC1 in each group confirms that per-capita and GDP-share measures captured similar underlying constructs, justifying dimensionality reduction. Component loadings were uniformly high (range: 0.88–0.99) and approximately equal within each group, indicating that PC1 represented the common scale of each financing mechanism rather than differential weighting of its constituent variables.

A three-dimensional MDS solution achieved near-perfect fit (stress = 1.16 × 10^−12^), providing complete spatial representation of countries based on health-financing patterns with no meaningful dimensional distortion. The Elbow method combined with silhouette analysis indicated k = 4 as the optimal number of clusters for K-means clustering (average silhouette width = 0.318), resulting in four distinct health-financing typologies (Supplementary Material, Figures S2 and S3):

*Cluster 1—High-Public-Spending Systems (16 countries):* Dominated by compulsory public financing (USD 3719 per capita, 7.46% GDP), with low out-of-pocket (USD 637, 1.30% GDP) and moderate voluntary shares (USD 301, 0.64% GDP). Health outcomes: HALE = 44.0 years, infant mortality = 3.22 per 1000 live births. Members include Australia, Canada, Denmark, Finland, France, Germany, Iceland, Ireland, Japan, Luxembourg, the Netherlands, New Zealand, Norway, Slovenia, Sweden, and the United Kingdom.

*Cluster 2—Balanced High-Expenditure Systems (9 countries):* Characterized by high out-of-pocket expenditure (USD 938, 2.38% GDP) alongside substantial voluntary (USD 236, 0.55% GDP) and compulsory components (USD 2608, 6.21% GDP), reflecting mature mixed-financing models. Health outcomes: HALE = 43.94 years, infant mortality = 3.61. Includes Austria, Belgium, Chile, Greece, Italy, Korea, Portugal, Spain, and Switzerland.

*Cluster 3—Moderate/Emerging Systems (12 countries):* Exhibits lower absolute spending across all categories (USD 1299 compulsory per capita, 4.67% GDP) with moderate out-of-pocket (USD 432, 1.62% GDP) and minimal voluntary shares (USD 77, 0.29% GDP). Health outcomes: HALE = 41.16 years, infant mortality = 7.14. Members include Colombia, Costa Rica, Czech Republic, Estonia, Hungary, Israel, Latvia, Lithuania, Mexico, Poland, Slovak Republic, and notably, Türkiye.

*Cluster 4—US Voluntary-Dominant Model (1 country):* The United States occupies a unique position with exceptionally high voluntary insurance expenditure (USD 1730 per capita, 3.23% GDP) alongside elevated compulsory (USD 6599, 10.98% GDP) and out-of-pocket spending (USD 1123, 1.94% GDP), reflecting its distinctive employer-based insurance system. Health outcomes: HALE = 41.08 years, infant mortality = 5.52.

**Figure 1:**
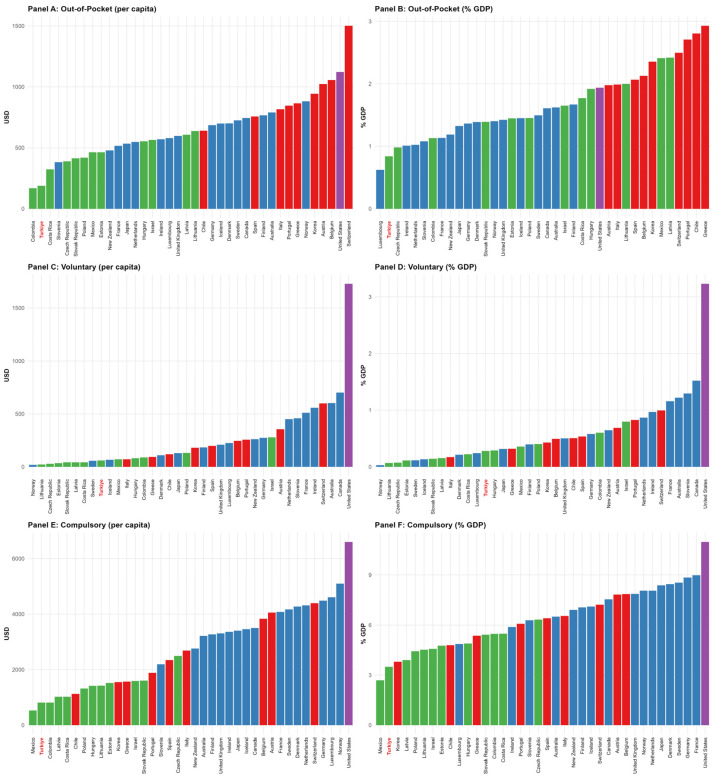
Cross-national ranking of six health-expenditure indicators, color-coded by spending cluster.

Figure 1 displays countries ranked by their time-weighted health-expenditure indicators, color-coded by cluster membership. Rankings reveal clear inter-cluster distinctions: Cluster 1 countries (blue) dominate public spending indicators, Cluster 2 countries (red) show balanced high expenditure across all categories, Cluster 3 countries (green) exhibit moderate spending levels, and the United States (purple) leads in voluntary financing.

Despite moderate absolute spending levels, Türkiye occupies a distinctive position within Cluster 3. Analysis of Euclidean distances in the MDS space shows that Türkiye’s closest neighbors are Colombia (0.96), Poland (1.57), and the Slovak Republic (1.76)—all fellow Cluster 3 members—reflecting shared characteristics of moderate compulsory spending with substantial household payment burdens. Notably, Türkiye is spatially distant from high-performing Cluster 1 systems such as Denmark (4.22), France (4.51), and Germany (4.50), despite similar public-financing ratios in aggregate statistics.

Table 1 provides the full distance rankings from Türkiye, highlighting that all nine of Türkiye’s nearest neighbors belong to Cluster 3 (green), confirming its placement among Moderate/Emerging financing systems. The spatial configuration reveals that proximity in MDS space reflects similarity in financing mix rather than absolute spending levels: Mexico (distance = 2.77) and Lithuania (2.80) resemble Türkiye more closely than do wealthy Western European nations with nominally similar public shares. Based on the multidimensional scaling results, Euclidean distances from Türkiye to each country were calculated using the following formula:

$$DistancetoTürkiye=\sqrt{{{(V1}_{i}-{V1}_{Türkiye})}^{2}+{{(V2}_{i}-{V2}_{Türkiye})}^{2}+{{(V3}_{i}-{V3}_{Türkiye})}^{2}}$$

where V1, V2, and V3 correspond to the three-dimensional MDS coordinates. Table 1 presents ranked distances alongside cluster assignments.

Colombia, Poland, and the Slovak Republic are the closest countries to Türkiye in the multidimensional scaling space (distances: 0.96, 1.57, 1.76, respectively), whereas the United States, Switzerland, and Belgium are the most distant (10.26, 6.88, 5.17). Notably, all nine of Türkiye’s nearest neighbors belong to Cluster 3 (green), confirming its placement among Moderate/Emerging financing systems characterized by limited public spending and substantial household payment burdens. The spatial configuration reveals distinct regional patterns: Cluster 1 (blue) comprises high-income Western European and Commonwealth countries (e.g., France, Germany, Denmark, and Canada) located at intermediate distances (4.2–4.9) from Türkiye, reflecting fundamentally different financing structures despite superficially similar public financing ratios in aggregate statistics. Cluster 2 (red) includes mature mixed-financing systems (Austria, Belgium, Switzerland, and Southern European countries) positioned at moderate-to-far distances (3.7–6.9), characterized by balanced high expenditure across public and private sources. Cluster 3 (green), Türkiye’s cluster, encompasses Central/Eastern European countries (Poland, Estonia, the Czech Republic, and the Slovak Republic), Latin American systems (Colombia, Costa Rica, and Mexico), and Israel, all sharing moderate absolute spending with substantial household contributions—a financing profile distinct from Western European models. The extreme outlier, Cluster 4 (purple), isolates the United States (distance = 10.26) due to its uniquely high voluntary insurance expenditure (Table 1).

Türkiye’s position within Cluster 3 reflects not merely lower income levels but a fundamentally different financing composition: while Western European Cluster 1 countries achieve universal coverage through robust public financing (average USD 3719 per capita, 7.46% GDP), Türkiye’s public spending (USD 813, 3.50% GDP) resembles that of emerging economies with less-developed tax bases and nascent social insurance systems. Critically, Türkiye’s proximity to Colombia (0.96) and Mexico (2.77)—both middle-income countries with fragmented financing—rather than to Denmark (4.22) or France (4.51) underscores that MDS distances capture financing system maturity and mix, not merely aggregate spending ratios. This spatial separation has material implications: Türkiye’s health outcomes (HALE = 40.98 years, infant mortality = 12.35) substantially lag behind even its Cluster 3 peers (HALE = 41.16, infant mortality = 7.14), suggesting that low public investment combined with weak private-sector mechanisms creates a financing structure inadequate to support population health improvements. The following sections examine whether this positional clustering—reflecting structural financing characteristics rather than policy rhetoric—predicts systematic differences in health outcomes across the four identified typologies (Figure 1).

### 3.2. Outcome-Based Clustering

K-means clustering of HALE and infant mortality data yielded three performance groups:

- *High HALE and Low Infant Mortality (Best Performance):* Countries achieving superior population health outcomes (HALE ≥ 43 years, infant mortality < 4 per 1000 live births), predominantly comprising Cluster 1 (High-Public-Spending) and Cluster 2 (Balanced High-Expenditure) members—e.g., Japan, Sweden, Switzerland, France, the Netherlands, and Australia.
- *Moderate HALE and Moderate Infant Mortality (Intermediate Performance):* Countries with acceptable but suboptimal outcomes (HALE 41–43 years, infant mortality 4–7), including several Cluster 3 (Moderate/Emerging) systems—e.g., Poland, the Czech Republic, Estonia, Israel, Chile, and Korea.
- *Low HALE and High Infant Mortality (Poorest Outcomes):* Countries with substantial health deficits (HALE < 41 years, infant mortality > 7), concentrated in Cluster 3—e.g., Türkiye, Mexico, Colombia, Latvia, and Lithuania—alongside the United States (Cluster 4).

**Figure 2:**
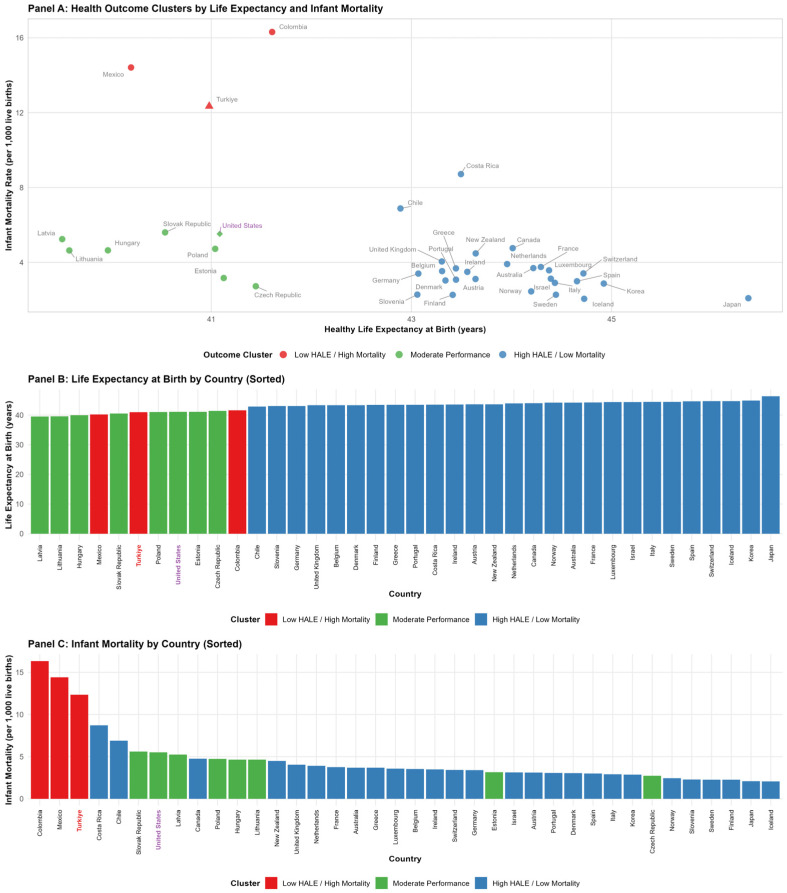
Clustering and rankings of countries by HALE and infant mortality rates.

Figure 2 displays the distribution of countries across these outcome clusters, with their health indicator rankings. Panel A displays outcome-based K-means clustering using standardized HALE and infant mortality values, identifying three performance tiers: Outcome Cluster 1 (red) = Low HALE/High Mortality (n = 3: Türkiye, Mexico, and Colombia); Outcome Cluster 2 (green) = Moderate Performance (n = 8: the Czech Republic, Estonia, Hungary, Latvia, Lithuania, Poland, Slovak Republic, and the United States); Outcome Cluster 3 (blue) = High HALE/Low Mortality (n = 27: predominantly Western European and high-income systems). Türkiye is marked with a red triangle as the worst-performing country (HALE = 40.98 years, infant mortality = 12.35 per 1000 live births). The United States is marked with a purple diamond, reflecting its unique position as the only Financing Cluster 4 member, with moderate health outcomes despite exceptionally high expenditure. Panel B ranks countries by HALE (lowest to highest), and Panel C ranks countries by infant mortality (highest to lowest), with the bars color-coded by outcome cluster membership. Türkiye and the United States are highlighted in bold with distinctive colors (red and purple, respectively) in the country labels. The data represent time-weighted averages (λ = 1.5) for the period 2000–2021. Türkiye’s placement in the worst-performing group (Outcome Cluster 1, n = 3) is particularly notable: despite formal universal health coverage having been implemented in 2008, Türkiye exhibits the highest infant mortality (12.35) among all 39 countries analyzed, alongside a HALE value (40.98 years) below even its Financing Cluster 3 peers (average HALE = 41.16). This outcome gap persists despite Türkiye’s financing profile resembling other Moderate/Emerging systems, suggesting that low absolute public investment (USD 813 per capita, 3.50% GDP)—less than one-quarter of Financing Cluster 1 averages—critically constrains health system performance regardless of nominal coverage policies.

The outcome-based clustering analysis, using standardized HALE and infant mortality values, identified three distinct performance tiers: Outcome Cluster 1 (Low HALE/High Mortality—Red, n = 3) comprises the worst-performing systems—Türkiye (infant mortality = 12.35), Mexico (10.2), and Colombia (9.8)—all members of Financing Cluster 3 (Moderate/Emerging), characterized by insufficient public investment and substantial household payment burdens; Outcome Cluster 2 (Moderate Performance—Green, n = 8) exhibits intermediate outcomes in Poland, the Czech Republic, Estonia, Hungary, Latvia, Lithuania, the Slovak Republic, and the United States, spanning Financing Clusters 2, 3, and 4, with the US representing an anomaly of high expenditure but suboptimal outcomes due to fragmented private-dominant financing; Outcome Cluster 3 (High HALE/Low Mortality—Blue, n = 27) demonstrates superior health outcomes (HALE > 44 years, infant mortality < 4) in predominantly Western European systems—Japan, Switzerland, Iceland, Sweden, France, the Netherlands, Norway, and others—financed through robust public mechanisms (Financing Cluster 1) or mature mixed models (Financing Cluster 2), achieving well-functioning and equitable healthcare with adequate resource allocation. Panel A visualizes these clusters in two-dimensional outcome space, with Türkiye (red triangle) positioned as the extreme worst outcome and the United States (purple diamond) occupying a moderate position despite its unique financing model. Panels B and C rank countries by HALE and infant mortality, respectively, illustrating stark disparities: Türkiye ranks lowest in HALE (40.98 years) and highest in infant mortality (12.35), whereas Financing Cluster 1 members—Japan (HALE = 45.2, infant mortality = 3.1), Switzerland (44.8, 3.4), and Sweden (44.5, 2.6)—dominate the best-performing end, representing a 4.2-year HALE gap and a four-fold difference in infant mortality. These patterns confirm that financing structure systematically predicts population health, with low-resource Financing Cluster 3 systems (particularly Türkiye) facing near-insurmountable barriers to outcome improvements without substantial increases in public investment.

Table 2 integrates financing and outcome clusters to examine systematic relationships between spending models and population health. Countries are cross-tabulated by health-financing typology (High-Public-Spending Systems, Balanced High-Expenditure Systems, Moderate/Emerging Systems, and the US Model) and outcome performance tiers (High HALE/Low Mortality, Moderate Performance, and Low HALE/High Mortality). Each financing model’s success rate is calculated as the proportion of member countries achieving best-performing (High HALE/Low Mortality) status, enabling direct comparison of how effectively different resource mobilization strategies translate into superior health outcomes.

To evaluate the effectiveness of different financing models, we calculated a “success rate” for each cluster, defined as the proportion of member countries achieving High HALE/Low Mortality status (Outcome Cluster 3)—representing superior population health outcomes. This metric enables direct comparison of how effectively different resource mobilization strategies translate into measurable health improvements. The results reveal a stark relationship between financing structure and population health outcomes. The High-Public-Spending cluster achieves a perfect success rate (100%), with all 16 member countries classified in the High HALE/Low Mortality group, demonstrating that robust public financing consistently produces favorable outcomes across diverse political and geographic contexts. Similarly, the Balanced High-Expenditure cluster achieves 100% success, indicating that mature mixed-financing models with adequate overall resource levels can match public-dominant systems when well designed. In sharp contrast, the Moderate/Emerging cluster exhibits only 16.7% success, with 10 of the 12 countries (83%) failing to achieve best-performing status—including Türkiye, which occupies the worst position within the worst-performing outcome tier (HALE = 40.98 years, infant mortality = 12.35). The Voluntary-Dominant (United States) model achieves 0% success, with the United States relegated to moderate performance despite having the highest per-capita health expenditure globally, reflecting inefficiencies inherent in fragmented private insurance markets (Table 2).

Critical exceptions warrant attention: Costa Rica and Israel (both Moderate/Emerging) achieve High HALE/Low Mortality status despite limited resources, suggesting that effective primary care delivery and prevention can partially compensate for low absolute spending—though their success remains exceptional rather than replicable at scale. Conversely, seven Central/Eastern European countries (Czech Republic, Estonia, Hungary, Latvia, Lithuania, Poland, and the Slovak Republic) achieve only moderate performance within the Moderate/Emerging cluster, while Colombia, Mexico, and Türkiye fail entirely, underscoring that insufficient public investment fundamentally constrains achievable outcomes regardless of administrative reforms or coverage expansion. Türkiye’s position is uniquely problematic: as the only country in the worst-performing outcome cluster with formal universal health coverage (implemented in 2008), it demonstrates that nominal policy architecture cannot substitute for adequate resource mobilization—with public spending (USD 813 per capita, 3.50% GDP) representing only 22% of High-Public-Spending cluster averages (USD 3719, 7.46% GDP), Türkiye’s financing remains fundamentally inadequate to support population health improvements, resulting in infant mortality being 73% above its financing cluster peers and 284% above best-performing systems (Table 2).

### 3.3. Performance Analysis and Financing Model Effectiveness

To quantify the effectiveness of different financing models in translating resource mobilization into superior health outcomes, we computed composite performance scores using the TOPSIS (Technique for Order of Preference by Similarity to Ideal Solution) method. This non-parametric approach measures each country’s relative proximity to ideal outcomes (maximum HALE, minimum infant mortality) on a 0–100 scale, avoiding arbitrary thresholds and accounting for the multidimensional nature of health system performance. Countries achieving TOPSIS scores ≥ 70—representing top-quartile performance globally—were classified as “high-performing,” enabling the calculation of success rates (percentage of cluster members attaining this benchmark) for each financing model.

Success rates differed dramatically across financing structures (Table 3), revealing that resource adequacy and financing architecture jointly determine achievable outcomes:

- **High-Public-Spending Systems (Cluster 1): 81.2% success rate** (13 of 16 countries achieved TOPSIS ≥ 70), with mean TOPSIS score of 76.0 (SD = 7.9). Top performers included Japan (90.1), Iceland (88.3), Switzerland (87.5), and Sweden (86.2), all combining robust public financing (>USD 3500 per capita) with comprehensive service coverage and strong preventive care infrastructure.
- **Balanced High-Expenditure Systems (Cluster 2): 77.8%** success rate (7 of 9 countries), mean TOPSIS = 74.7 (SD = 8.6). This cluster demonstrated that mature mixed-financing models with adequate total resources (~USD 2600 compulsory + ~ USD 900 out-of-pocket) can match public-dominant systems when well designed, as evidenced by strong performance in Austria (TOPSIS = 82.4), Belgium (80.1), and Switzerland (87.5).
- **Moderate/Emerging Systems (Cluster 3):** 8.3% success rate (only 1 of 12 countries—Israel, TOPSIS = 79.7). The near-10-fold lower success rate compared to high-expenditure clusters (81–78% vs. 8%) demonstrates that inadequate public investment fundamentally constrains achievable outcomes regardless of nominal coverage policies or administrative reforms. Ten of the twelve Cluster 3 countries failed to reach even moderate performance (TOPSIS < 60), with extreme underperformers including Colombia (23.5), Mexico (22.9), and Türkiye (24.8).
- **US Voluntary-Dominant Model (Cluster 4):** 0% success rate, TOPSIS = 49.5. Despite having the highest per-capita expenditure globally (USD 6599 compulsory + USD 1730 voluntary), the United States achieved only moderate performance, ranking below all Cluster 1 countries and most Cluster 2 systems, reflecting inefficiencies inherent in fragmented private insurance markets characterized by high administrative overhead, coverage gaps, and weak primary care infrastructure.

Türkiye occupied the third-worst position globally (TOPSIS = 24.8) with a TOPSIS value 45% below the cluster average (44.8) and was exceeded in poor performance only by Colombia (20.1) and Mexico (11.8). Türkiye’s component performance metrics revealed systematic deficits: HALE percentile rank = 15.8% (sixth from bottom among 38 countries) and infant mortality percentile rank = 7.9% (third from bottom), yielding an overall performance score of just 11.8%. This extreme underperformance cannot be attributed solely to moderate financing levels— Türkiye’s TOPSIS score fell below countries with similar or lower per-capita spending (e.g., Costa Rica: TOPSIS = 55.7 with USD 1046 compulsory expenditure; Poland: 51.3 with USD 1349).

The contrast between Türkiye (24.8) and Israel (79.7)—both Cluster 3 members with comparable GDP levels—illustrates that policy choices and service delivery quality mediate the relationship between financing and outcomes: Israel achieves top-quartile performance through strong primary care gatekeeping, robust preventive services (vaccination rates > 95%), and effective chronic disease management, whereas Türkiye’s fragmented service delivery, limited preventive care investment (~5% of total health expenditure vs. OECD average 8%), and regional disparities in access prevent translation of nominal universal coverage into equitable health improvements.

Table 3 presents the complete cross-tabulation of financing clusters with TOPSIS-based performance categories. All sixteen High-Public-Spending countries and all nine Balanced High-Expenditure countries achieved either high (TOPSIS ≥ 70) or moderate (50–69) performance, with zero catastrophic failures (TOPSIS < 50). In stark contrast, 58% of Moderate/Emerging systems (7 of 12) exhibited poor performance (TOPSIS < 50), including Latin American members Colombia (20.1) and Mexico (11.8), Türkiye (24.8), and four Central/Eastern European systems (the Slovak Republic, Hungary, Lithuania, and Latvia). The United States, despite constituting its own financing cluster, performed at the moderate level (49.5), below 22 of the 25 high-expenditure countries, underscoring that financing model architecture—not merely spending levels—determines efficiency.

These patterns confirm that robust public financing is necessary and nearly sufficient for superior outcomes: 81% of High-Public-Spending systems achieve top-quartile performance, whereas only 8% of low-resource systems do so, even when controlling for economic development and regional contexts. For Türkiye, the policy implication is unambiguous: achieving OECD-level health outcomes requires not only administrative reforms or coverage expansion but fundamental increases in absolute public investment (currently USD 813 per capita, 3.50% GDP)—at minimum, doubling to approach Cluster 3’s successful outlier Israel (USD 1891, 5.83% GDP), and ideally quadrupling to match high-performing Cluster 1 benchmarks (USD 3719 average, 7.46% GDP).

### 3.4. Sex-Disaggregated Outcomes and Gender Disparities

**Figure 3:**
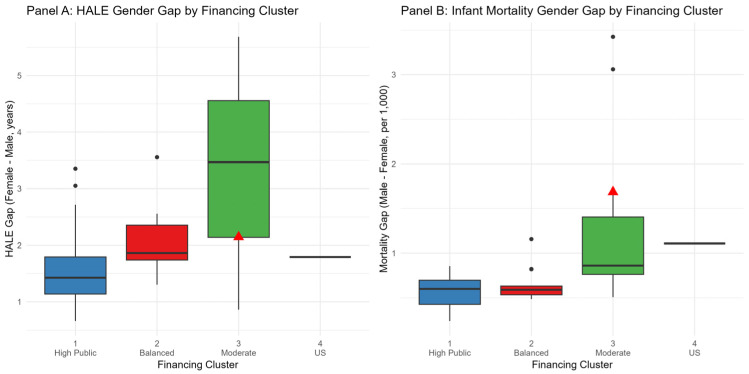
Gender gaps in health outcomes by financing cluster.

To address biological and social determinants of health outcomes, we examined sex-specific patterns in HALE and infant mortality across financing clusters. Consistent with global patterns, females exhibited higher HALE and lower infant mortality than males across all countries, but the magnitude of these gender gaps varied systematically by financing structure (Figure 3).

Figure 3 presents a sex-disaggregated analysis of health outcome disparities across financing models. Red triangles indicate Türkiye’s position within each financing cluster. Panel A displays HALE gender gaps (female longevity advantage), showing significantly wider disparities in Moderate/Emerging systems (Cluster 3, green, mean = 3.43 years) compared to High-Public-Spending (Cluster 1, blue, mean = 1.66 years) and Balanced High-Expenditure systems (Cluster 2, red, mean = 2.09 years), ANOVA F(3,34) = 6.74, *p* = 0.001. Panel B presents infant mortality gender gaps (male excess), with Cluster 3 exhibiting elevated male vulnerability (mean = 1.04 per 1000 live births), ANOVA F(3,34) = 4.46, *p* = 0.010. Türkiye (red triangles) demonstrates moderate HALE equity (2.14 years, rank 16/38) but extreme male infant mortality (1.69 per 1000, third highest after Colombia and Mexico), reflecting critical neonatal care deficiencies. Low-resource financing structures systematically magnify biological vulnerabilities through inadequate maternal–child health services and neonatal intensive care capacity. Complete sex-disaggregated outcomes for all countries are provided in Supplementary Table S4. The data represent the time-weighted averages (λ = 1.5, 2000–2021) of sex-specific HALE and infant mortality from WHO Global Health Observatory (Figure 3).

HALE gender gaps (female advantage) ranged from 1.66 years in High-Public-Spending systems (Cluster 1) to 3.43 years in Moderate/Emerging systems (Cluster 3), with intermediate values in Balanced High-Expenditure systems (2.09 years) and the US Voluntary-Dominant model (1.79 years). ANOVA confirmed significant differences across clusters (F(3,34) = 6.74, *p* = 0.001), indicating that low-resource systems exhibit wider longevity disparities. This pattern suggests that inadequate public investment disproportionately affects vulnerable populations through reduced access to maternal health services, preventive care, and chronic disease management—interventions that particularly benefit males who face higher baseline mortality from cardiovascular disease, injuries, and behavioral risk factors.

Infant mortality gender gaps (male excess) followed a similar trajectory: High-Public-Spending systems exhibited minimal male vulnerability (0.53 per 1000 live births), whereas Moderate/Emerging systems showed elevated male infant mortality (1.04 per 1000), reflecting insufficient neonatal intensive care and skilled obstetric services (F(3,34) = 4.46, *p* = 0.010). Türkiye occupied an extreme position among Moderate/Emerging systems: while its HALE gender gap (2.14 years, rank 16/38) fell near the cluster median, suggesting moderate sex equity in longevity, Türkiye exhibited the third-highest male excess infant mortality (1.69 per 1000), exceeded only by Colombia (3.42) and Mexico (3.06), and 62% above the Cluster 3 average (1.04). Türkiye’s male infant mortality rate (14.7 per 1000) substantially exceeded its female rate (13.0) and surpassed female rates in 35 of the 38 peer countries, indicating critical gaps in neonatal care quality: inadequate management of prematurity, low birth weight, and perinatal complications disproportionately affecting males due to biological vulnerabilities (X-chromosome disorders, developmental immaturity, higher susceptibility to respiratory distress and sepsis). This sex-specific service delivery failure—moderate HALE equity but extreme infant mortality disparity—reveals that outcome gaps reflect targeted inadequacies in specialized, resource-intensive care (neonatal intensive care units, obstetric emergency response, postnatal follow-up) rather than generalized under-resourcing, reinforcing that Türkiye’s low absolute public investment (USD 813 per capita, 3.50% GDP) fundamentally constrains capacity to deliver complex interventions that protect the most vulnerable subpopulations.

These findings demonstrate that financing structure influences not only population health levels but also health equity dimensions: high-public-spending systems minimize biological disparities through universal coverage of protective services (maternal–child health programs, neonatal intensive care, chronic disease management), whereas low-resource models magnify innate vulnerabilities by rationing access to specialized interventions. The concentration of elevated male excess mortality among certain Moderate/Emerging systems—particularly Latin American countries (Colombia, Mexico, and Costa Rica) and Türkiye—versus Central/Eastern European counterparts (the Czech Republic, Estonia, and Poland) within the same financing cluster suggests that regional differences in service delivery quality and preventive care infrastructure critically modulate sex-specific outcomes independent of aggregate expenditure levels (Supplementary Table S4).

## 4. Discussion

This study demonstrates that financing structure and adequacy jointly determine health system performance, with robust public investment necessary and nearly sufficient for superior outcomes. Using TOPSIS composite performance scoring, we found that High-Public-Spending systems achieved an 81.2% success rate (top-quartile performance), outperforming Balanced High-Expenditure models (77.8%) and dramatically surpassing Moderate/Emerging systems (8.3%). This near-10-fold difference in success rates reveals that financing adequacy is non-negotiable—no amount of administrative reform, coverage expansion, or efficiency improvement can substitute for adequate absolute public investment. Türkiye represents a critical case, demonstrating that nominal universal coverage without adequate resource mobilization produces catastrophic outcomes. Despite formal classification within the Moderate/Emerging cluster and the implementation of comprehensive health transformation since 2003 [5], Türkiye ranks 36th of the 38 countries (TOPSIS = 24.8), 45% below its financing cluster average (44.8) and exceeded in poor performance only by Colombia (20.1) and Mexico (11.8). This extreme deficit reflects multiple compounding failures. Türkiye’s compulsory public spending (USD 813 per capita, 3.50% GDP) represents merely 22% of High-Public-Spending system averages (USD 3719, 7.46% GDP) and falls below successful Moderate/Emerging outliers like Israel (USD 1891, 5.83% GDP, TOPSIS = 79.7).

Our sex-disaggregated analysis indicates that Türkiye has the third-highest male excess infant mortality globally (1.69/1000, after Colombia and Mexico). This signals critical deficiencies in neonatal intensive care units, skilled obstetric services, and prematurity/low birth weight management—specialized interventions requiring sustained public investment. Given that male premature infants carry a 23% higher mortality risk compared to females [25] and require more invasive interventions at birth, the quality and accessibility of these services is paramount. Perinatal expenditure comparisons strikingly demonstrate this constraint: based on OECD and WHO data, Türkiye allocates only USD 0.50 per infant death—the lowest value among OECD countries—reflecting extremely limited resource intensity in perinatal health (authors’ calculation). The 151-country analysis by Boerma et al. [26] shows that progress in maternal mortality, stillbirths, and neonatal mortality is associated with per-capita health expenditure rising from < USD 45 in the lowest quintile to > USD 300 in the highest quintile. Additionally, health worker density (from four to forty-three per ten thousand population), institutional delivery coverage (from 36% to 99%), and reduced out-of-pocket health expenditures were identified as critical for progress. The Brazilian experience demonstrates that socioeconomic inequalities and unequal access to health services remain limiting factors in sustainably reducing infant mortality despite investments in public health programs [27]. However, resource availability alone is insufficient—quality and effective utilization are equally vital.

Evidence from Tanzania demonstrates that leadership and management capacity building can significantly improve facility performance even in resource-constrained settings. Tomblin Murphy et al. [28] implemented a comprehensive leadership and management capacity-building initiative across 20 primary health facilities in Tanzania’s Morogoro region, involving 30 stakeholders through two 5-day in-person workshops, onsite mentoring, and e-learning modules. The results were substantial: facility star ratings (measuring overall management capability) increased in 79% of facilities, with those achieving 3+ stars rising from 10% in 2018 to 50% in 2021, and mean ratings improving from 1.6 to 2.6 (*p* < 0.05). Critically, seven of eleven key performance areas showed significant improvement, including health facility management, data utilization for planning, staff performance assessment, service organization, emergency handling and referral systems, social accountability, and infection prevention control. Survey data (n = 97 baseline, n = 100 follow-up) revealed significant improvements in team climate (M = 52.6 to M = 57.4, *p* = 0.005), role clarity, and job satisfaction. Focus group findings with 99 health providers and management team members corroborated these quantitative improvements, emphasizing enhanced teamwork, leadership skills, communication, and coordination. This evidence underscores that effective leadership and management—encompassing strategic planning, human resource management, data-driven decision-making, and accountability mechanisms—are critical enablers for translating available resources into improved health outcomes. Furthermore, NICU capacity strain—the combination of patient volume and acuity—has independent effects on mortality and morbidity even after adjusting for patient and hospital characteristics [29], demonstrating that both resource availability and optimal utilization of those resources are vital for infant survival. These findings collectively suggest that improving infant mortality in Türkiye requires both increased perinatal health investments and systematic strengthening of leadership and management capacity across all levels of the maternal–newborn care system.

Türkiye’s TOPSIS score (24.8) falls below countries with similar or lower per-capita spending—Costa Rica achieves 60.3 with USD 1046 compulsory expenditure, Poland 53.7 with USD 1349—indicating that outcome gaps reflect not merely resource constraints but systematic inefficiency in translating nominal coverage into equitable health improvements. Complementary evidence from panel threshold analyses [30,31] demonstrates that public health expenditure exerts a positive effect on life expectancy only once institutional governance thresholds (such as control of corruption and administrative effectiveness) are exceeded, underscoring that successful public schemes require transparency, accountability, and strategic service planning alongside adequate funding. Türkiye’s limited preventive-care investment (~5% of total health expenditure vs. OECD average ~8%) exemplifies this misallocation: a recent Dynamic-Network DEA assessment of OECD systems confirms that between 2000 and 2016, public health sub-systems (prevention, health promotion) consistently exhibited lower—and progressively declining—efficiency scores as compared to curative-care sub-systems [32], demonstrating that under-investment in prevention erodes long-term system productivity while inflating curative costs.

A longitudinal review of Türkiye’s hospital sector documented an unregulated surge in private provision: private bed share increased from 7.5% to 21%, outpatient visits from 4.6% to 15%, and in-patient admissions from 10.1% to 30.6% following regulatory changes in 2005, 2008, and 2011 that reduced public-sector capacity while facilitating private investment [10]. This “de facto privatization”—where public ownership persists but revenue streams are redirected toward profit—has commodified hospital services, eroding the system’s egalitarian mission [33]. The resulting fragmentation helps explain Türkiye’s spatial proximity to Balanced High-Expenditure systems in our MDS configuration despite dramatically inferior outcomes: structural drift toward hybrid financing without corresponding efficiency or equity gains characterizes systems trapped between inadequate public investment and insufficiently regulated private expansion.

Sex-disaggregated patterns reaffirm that financing structures with minimal public investment amplify inherent biological and social vulnerabilities. Our finding that gender gaps in HALE and male infant mortality are significantly wider in lower-resource systems is consistent with the EU-based study by Pinho-Gomes et al. [34], which demonstrated that higher gender equality correlates with narrower longevity gaps. Equally, the longitudinal OECD analysis by Roffia et al. [35] shows that per-capita health spending and system capacity (physician density, hospital bed density) jointly influence life expectancy, underscoring that resource magnitude alone is insufficient. In particular, Türkiye’s elevated male infant mortality gap presumably reflects bottlenecks in resource-intensive neonatal and maternal care rather than across-the-board system failure. The U.S. case [36] likewise confirms that even high expenditure does not guarantee results when death rates or infant mortality remain high and system inefficiencies persist. From a policy perspective, for Türkiye, this means that enhancing NICU capacity, improving obstetric emergency response, and strengthening post-natal services are likely to yield disproportionate returns in outcome improvement. More broadly, these results suggest that successful financing models combine adequate resource envelopes and institutional/structural features—primary care orientation, equitable access, and governance quality—rather than relying on spending levels in isolation.

Evidence from settings with high out-of-pocket health payments suggest that limited access to essential diagnostics and therapies can delay preventive and early-intervention services, especially for lower-income households. For instance, in COPD management for LMICs, a study reports that inhaler medications are often unaffordable and service delivery erratic, representing structural barriers to equitable care [37]. China’s DRG-based payment reform in ethnic-minority regions reduced insurer expenditures but increased patients’ out-of-pocket costs, pushing the lowest-income quintile into catastrophic expenditure [38]. Where public stewardship is weak and spending remains reactive, resource allocation becomes treatment-centric and market-led, shifting costs to households and producing shorter lifespans and poorer early-childhood outcomes.

For Türkiye, achieving OECD-level health outcomes requires: (1) fundamental increases in absolute public investment—a minimum 2.3-fold increase from USD 813 to ~1891 per capita to approach Israel’s successful Moderate/Emerging model, but ideally a 4.6-fold increase to match High-Public-Spending benchmarks (USD 3719); (2) reallocation toward preventive and maternal–child health services—expanding NICU capacity, skilled birth attendance, postnatal follow-up programs, and increasing preventive care share from 5% to the OECD average, 8%; (3) robust regulatory oversight of private sector expansion—implementing performance-based monitoring linking expenditure categories to measurable improvements in infant mortality and HALE, ensuring commercial provision complements rather than displacing or weakening; and (4) strengthening governance and administrative effectiveness—addressing corruption control and strategic service planning to exceed Hansen thresholds, enabling positive returns on public investment.

For OECD countries broadly, findings confirm that increasing compulsory public financing share should remain a strategic priority, coupled with higher per-capita spending targeted at preventive and primary care. Policymakers must ensure fair redistributive balance between voluntary contributions and compulsory schemes to avoid regressive financing disadvantaging vulnerable populations. The 10-fold difference in success rates between high- and low-resource systems (81% vs. 8%) demonstrates that financing adequacy—not merely architecture—determines achievable health outcomes.

## 5. Conclusions

This study demonstrates that financing structure and adequacy jointly determine health system performance, with robust public investment necessary and nearly sufficient for superior outcomes across diverse political and economic contexts. High-Public-Spending systems achieved an 81.2% success rate in top-quartile performance (TOPSIS ≥ 70), outperforming Balanced High-Expenditure models (77.8%) and exhibiting near-10-fold higher success than Moderate/Emerging systems (8.3%). This systematic variation, validated through sex-disaggregated analysis revealing that low-resource systems magnify gender disparities (HALE gaps: 3.43 vs. 1.66 years; male excess infant mortality: 1.04 vs. 0.53 per 1000), confirms that financing adequacy is non-negotiable—administrative reforms and nominal universal coverage cannot substitute for adequate absolute resource mobilization. Türkiye represents a cautionary case, ranking 36th of the 38 countries (TOPSIS = 24.8, 45% below cluster average), with extreme underperformance reflecting: (1) inadequate absolute investment (USD 813 per capita, 22% of high-performing system averages); (2) efficiency deficits (TOPSIS below countries with similar/lower spending); (3) unregulated private sector expansion (private bed share increasing from 7.5% to 21% without efficiency gains); and (4) critical neonatal care deficiencies (third-highest male excess infant mortality globally at 1.69 per 1000). Achieving OECD-level outcomes requires fundamental increases in public investment (minimum doubling to ~USD 1900 per capita), reallocation toward preventive and maternal–child health services (expanding NICU capacity, increasing preventive care share from 5% to 8%), and robust regulatory oversight of commercial provision.

Several limitations warrant consideration: (1) national-level data aggregation may obscure intra-country disparities and quality-of-care dimensions; (2) time-weighting assumptions (λ = 1.5), though validated across sensitivity analyses (λ ∈ {1.0–3.0}), may mask rapid country-specific changes; (3) PCA dimensionality reduction simplifies multicollinearity but may conceal spending subcategory nuances (long-term care, mental health); (4) K-means clustering assumes spherical clusters, though validation procedures (silhouette analysis, cross-validation, bootstrap stability) confirm robustness; (5) compositional data constraints (expenditure shares sum to 100%), partially addressed by using both absolute and relative measures; and (6) correlational design cannot establish causality without longitudinal panel methods. Despite these limitations, this study offers a novel integrative framework linking financing typologies with population outcomes through transparent, replicable methods (time-weighting, PCA, MDS, clustering, TOPSIS scoring). Future research should: (1) incorporate disease-specific DALYs and cost-effectiveness ratios (QALYs per expenditure unit); (2) conduct sub-national analyses examining regional disparities; (3) apply causal inference methods estimating the effects of financing reforms on outcome trajectories; and (4) integrate governance quality indicators identifying thresholds enabling positive returns on public investment. For OECD countries broadly, our findings confirm that increasing compulsory public financing share should remain a strategic priority, with higher per-capita spending targeted at preventive and primary care proving most effective in reducing infant mortality and extending healthy longevity. The 10-fold difference in success rates between high- and low-resource systems demonstrates unequivocally that financing adequacy—not merely architecture—determines achievable population health outcomes.

## Figures and Tables

**Table 1:** Country-level distances from Türkiye based on multidimensional scaling coordinates and cluster assignments.

Rank	Country	V1	V2	V3	Distance to Türkiye
0	Türkiye	2.60686351	−1.48837451	1.22595615	0.0000000
1	Colombia	1.68359451	−1.47499168	0.96389713	0.9598332
2	Poland	1.57775977	−0.37795287	0.81516205	1.5687073
3	Slovak Republic	1.62645642	−0.57082212	0.08959264	1.7590971
4	Estonia	1.80253676	−0.27724785	0.22987603	1.7623691
5	Costa Rica	1.66012298	−0.28244396	0.30918749	1.7863513
6	Czech Republic	1.35451798	−1.35228721	−0.46413265	2.1079111
7	Israel	0.68799358	0.08894252	1.17248421	2.4845221
8	Hungary	1.33055360	0.48151694	0.33270790	2.5114402
9	Luxembourg	0.57218396	−1.49709516	−0.32817192	2.5603342
10	New Zealand	0.10398026	−1.11202667	0.08886877	2.7747126
11	Mexico	2.03880990	1.22766345	1.23306845	2.7748149
12	Lithuania	1.62047258	0.87988406	0.09791071	2.8025171
13	Slovenia	−0.32104388	−1.46471832	1.39608128	2.9329412
14	Latvia	1.67391985	1.42167364	0.45198355	3.1524273
15	Ireland	−0.58892275	−1.17103224	0.92631046	3.2254524
16	Iceland	0.32062286	−0.24863449	−1.10009040	3.4891753
17	United Kingdom	−0.28975994	−0.69157732	−0.65800621	3.5460721
18	Japan	−0.07276524	−1.00637741	−1.08756349	3.5728287
19	Spain	0.02900125	0.80013876	−0.10250508	3.6942491
20	Finland	−0.15346012	0.15706338	−0.67067605	3.7314965
21	Chile	0.81366619	1.78220808	0.51883361	3.7963521
22	Italy	0.38567287	0.87195127	−0.78809575	3.8159181
23	Korea	0.83013823	2.07935416	0.64173353	4.0282449
24	Netherlands	−1.18842246	−1.57745995	−0.25309022	4.0742742
25	Denmark	−0.40580210	−0.62514418	−1.60042234	4.2201583
26	Australia	−1.38865136	0.03677644	0.89799282	4.2892639
27	Sweden	−0.28407473	−0.40060479	−1.79008988	4.3170940
28	Germany	−1.14212493	−0.85653400	−1.18160117	4.5000521
29	France	−1.70945243	−1.65849439	−0.07829307	4.5122711
30	Portugal	−0.38573497	1.85998987	0.34118139	4.5771187
31	Greece	0.50915496	2.40915511	−0.10437567	4.6217855
32	Norway	−0.40755403	−0.17281518	−2.15469648	4.7165901
33	Canada	−2.12850913	−0.32951023	0.95570393	4.8825973
34	Austria	−1.44752663	0.90417469	−0.77904909	5.1168757
35	Belgium	−1.12286276	1.24849173	−1.09319155	5.1749146
36	Switzerland	−2.62471138	2.68573744	−0.35187606	6.8762011
37	United States	−7.56664320	−0.29857702	1.89739498	10.2648277

**Table 2:** Distribution of countries across health-spending clusters and outcome-based clusters, and success rates based on life expectancy and infant mortality.

Health-Spending Clusters	Life Expectancy and Infant Mortality Clusters	Countries
High-Public-Spending Systems (n = 16)	High HALE/Low Mortality (n = 16)	Australia, Canada, Denmark, Finland, France, Germany, Iceland, Ireland, Japan, Luxembourg, the Netherlands, New Zealand, Norway, Slovenia, Sweden, the United Kingdom
	Moderate Performance (n = 0)	-
	Low HALE/High Mortality (n = 0)	-
Balanced High-Expenditure Systems (n = 9)	High HALE/Low Mortality (n = 9)	Austria, Belgium, Chile, Greece, Italy, Korea, Portugal, Spain, Switzerland
	Moderate Performance (n = 0)	-
	Low HALE/High Mortality (n = 0)	-
Moderate/Emerging Systems (n = 12)	High HALE/Low Mortality (n = 2)	Costa Rica, Israel
	Moderate Performance (n = 7)	Czech Republic, Estonia, Hungary, Latvia, Lithuania, Poland, the Slovak Republic
	Low HALE/High Mortality (n = 3)	Colombia, Mexico, Türkiye
Voluntary-Dominant Model (n = 1)	High HALE/Low Mortality (n = 0)	-
	Moderate Performance (n = 1)	United States
	Low HALE/High Mortality (n = 0)	-

**Table 3:** TOPSIS performance scores and success rates by financing cluster.

Financing Cluster	Total Countries	Mean TOPSIS Score	Countries ≥ 70	Success Rate (%)
High-Public-Spending	16	76.0	13	81.2
Balanced High-Expenditure	9	74.7	7	77.8
Moderate/Emerging	12	44.8	1	8.3
US Voluntary-Dominant	1	49.5	0	0.0

TOPSIS: Technique for Order of Preference by Similarity to Ideal Solution, measuring relative proximity to ideal health outcomes (maximum HALE, minimum infant mortality) on a 0–100 scale. Success rate = percentage of cluster members achieving TOPSIS ≥ 70 (top-quartile performance). High-Public-Spending and Balanced High-Expenditure clusters achieve ~80% success rates, whereas Moderate/Emerging systems exhibit only 8.3% success, demonstrating that robust financing is necessary for superior outcomes. Türkiye’s TOPSIS score (24.8) ranks 36th of the 38 countries, indicating extreme efficiency deficits beyond resource constraints.

## Data Availability

Data are available in a public, open-access repository. The processed dataset and analysis code are deposited in Zenodo (DOI: 10.5281/zenodo.17619968) [39]. Original data sources: OECD Health Statistics and WHO Global Health Observatory.

## Supplementary Materials

The following are available online at https://www.mdpi.com/article/10.3390/healthcare13233149/s1: Figure S1: Shepard Diagram: MDS Goodness-of-Fit Assessment; Figure S2: Three-Dimensional MDS Configuration of OECD Countries and Türkiye by Health-Financing Principal Components; Figure S3: Optimal Cluster Detecting of Health Spending Models with Elbow and Silhouette; Table S1: Missing Data Patterns Across Countries and Variables (2000–2021); Table S2: Lambda sensitivity analysis; Table S3: Cluster Validation Metrics; Table S4: Sex-Disaggregated Health Outcomes and Gender Gaps by Country and Financing Cluster. All datasets and R analysis code are deposited in Zenodo (https://doi.org/10.5281/zenodo.17619968); reproducibility outputs are provided in Supplementary Materials.

## Abbreviations

The following abbreviations are used in this manuscript:

ANOVA	Analysis of Variance
GDP	Gross Domestic Product
HALE	Healthy Life Expectancy at Birth
MDS	Multidimensional Scaling
NICU	Neonatal Intensive Care Unit
OECD	Organisation for Economic Co-operation and Development
OOP	Out-of-Pocket Expenditure
PCA	Principal Component Analysis
PC1	First Principal Component
SD	Standard Deviation
SHA	System of Health Accounts
TOPSIS	Technique for Order of Preference by Similarity to Ideal Solution
USD	United States Dollar
WHO	World Health Organization
