# Supplementary material for: Evaluating Health Financing Typologies Through Healthy Life Expectancy and Infant Mortality: Evidence from OECD Countries and Türkiye
Source: Healthcare (Basel). 2025 Dec 2;13(23):3149. doi: 10.3390/healthcare13233149 (PMC12691894; doi:10.3390/healthcare13233149)
Supplement: Supplementary file 1 [file healthcare-13-03149-s001.zip › healthcare-3954741-supplementary.pdf]

Supplementary Material

Missing Value Analysis

The final analytical dataset comprised 1,373 country-year observations across 38 OECD countries and Türkiye from 2000 to 2021 (Table S1).

Table S1. Missing Data Patterns Across Countries and Variables (2000-2021)

Country	Variable	Missing Count	Missing Years	First Missing	Last Missing
Belgium	Out-of-Pocket (% of GDP)	6	2000, 2001, 2002	2000	2002
Belgium	Out-of-Pocket (Per Capita)	6	2000, 2001, 2002	2000	2002
Belgium	Voluntary (% of GDP)	6	2000, 2001, 2002	2000	2002
Belgium	Voluntary (Per Capita)	6	2000, 2001, 2002	2000	2002
Costa Rica	Compulsory (% of GDP)	4	2000, 2001, 2002, 2003	2000	2003
Costa Rica	Compulsory (Per Capita)	4	2000, 2001, 2002, 2003	2000	2003
Costa Rica	Out-of-Pocket (% of GDP)	4	2000, 2001, 2002, 2003	2000	2003
Costa Rica	Out-of-Pocket (Per Capita)	4	2000, 2001, 2002, 2003	2000	2003
Costa Rica	Voluntary (% of GDP)	4	2000, 2001, 2002, 2003	2000	2003
Costa Rica	Voluntary (Per Capita)	4	2000, 2001, 2002, 2003	2000	2003
Czech Republic	Voluntary (% of GDP)	6	2000, 2001, 2002	2000	2002
Czech Republic	Voluntary (Per Capita)	6	2000, 2001, 2002	2000	2002
Greece	Out-of-Pocket (% of GDP)	3	2000, 2001, 2002	2000	2002
Greece	Out-of-Pocket (Per Capita)	3	2000, 2001, 2002	2000	2002
Greece	Voluntary (% of GDP)	3	2000, 2001, 2002	2000	2002
Greece	Voluntary (Per Capita)	3	2000, 2001, 2002	2000	2002
Korea	Infant Mortality (Both Sexes)	4	2000, 2001, 2003, 2004	2000	2004
New Zealand	Infant Mortality (Both Sexes)	1	2021	2021	2021
New Zealand	Out-of-Pocket (% of GDP)	2	2003	2003	2003
New Zealand	Out-of-Pocket (Per Capita)	2	2003	2003	2003
New Zealand	Voluntary (% of GDP)	2	2003	2003	2003
New Zealand	Voluntary (Per Capita)	2	2003	2003	2003
Poland	Voluntary (% of GDP)	4	2000, 2001	2000	2001
Poland	Voluntary (Per Capita)	4	2000, 2001	2000	2001
Slovak Republic	Voluntary (% of GDP)	8	2000, 2001, 2002, 2003	2000	2003
Slovak Republic	Voluntary (Per Capita)	8	2000, 2001, 2002, 2003	2000	2003
Slovenia	Out-of-Pocket (% of GDP)	3	2000, 2001, 2002	2000	2002
Slovenia	Out-of-Pocket (Per Capita)	3	2000, 2001, 2002	2000	2002
Slovenia	Voluntary (% of GDP)	3	2000, 2001, 2002	2000	2002
Slovenia	Voluntary (Per Capita)	3	2000, 2001, 2002	2000	2002
Sweden	Voluntary (% of GDP)	2	2000	2000	2000
Sweden	Voluntary (Per Capita)	2	2000	2000	2000
Türkiye	Infant Mortality (Both Sexes)	2	2000, 2001	2000	2001

HALE = Healthy Life Expectancy at birth; OOP = Out-of-pocket expenditure; GDP = Gross Domestic Product. Missing data predominantly occurred in early years (2000-2003) due to delayed implementation of System of Health Accounts (SHA) reporting standards. Twenty-seven countries (71%) had complete data across all variables and years. Variables with zero missing values: HALE (both sexes, male, female), infant mortality (both sexes, male, female). Most affected variables: Voluntary expenditure (per capita and % GDP): 38 missing observations (2.76%), primarily in Belgium, Costa Rica, Czech Republic, Greece, New Zealand, Poland, Slovak Republic, Slovenia, and Sweden.

Overall data completeness was high, with 95% of all data points present. Missing data were concentrated in health expenditure variables during the early 2000s, reflecting differences in national reporting timelines following the implementation of the System of Health Accounts (SHA) framework. All HALE and sex-disaggregated infant mortality variables achieved complete coverage. The pattern of missingness was non-random but systematic, occurring primarily in voluntary and out-of-pocket expenditure measures for specific countries during 2000-2003, which preceded full adoption of standardized health accounting practices in these jurisdictions (Table S1).

Lambda Sensitivity Analysis

To determine the optimal exponential weighting parameter (λ), we conducted a comprehensive sensitivity analysis across five values: $\lambda \in \{1.0, 1.5, 2.0, 2.5, 3.0\}$. For each λ value, we calculated time-weighted country-level averages, performed principal component analysis on the three financing groups, and applied K-means clustering (k=3) to the resulting component space. Clustering quality was assessed using the average silhouette coefficient and between-cluster to within-cluster sum-of-squares ratio. Predictive validity was evaluated by regressing health outcomes (HALE and infant mortality) on cluster membership and calculating eta-squared (η^2) as a measure of variance explained. The optimal λ balances clustering stability with outcome prediction accuracy.

Table S2. Lambda sensitivity analysis

λ Value	Silhouette Coefficient	Between/ Within SS Ratio	HALE η^2	Infant Mortality η^2	Weight Ratio*
**1.0**	0.313	1.276	0.488	0.278	2.7:1
**1.5**	0.301	1.198	0.489	0.270	4.5:1
**2.0**	0.294	1.122	0.346	0.180	7.4:1
**2.5**	0.286	1.046	0.349	0.174	12.2:1
**3.0**	0.284	0.999	0.222	0.196	20.1:1

*Weight ratio represents the relative weight assigned to the most recent year (2021) compared to the earliest year (2000). Silhouette coefficient ranges from -1 to +1, with higher values indicating better-defined clusters. Between/Within SS ratio measures cluster separation, with values >1 indicating greater between-cluster than within-cluster variance. Eta-squared (η^2) represents the proportion of outcome variance explained by cluster membership. Bold row indicates the selected optimal value (λ=1.5), which maximized HALE prediction while maintaining high clustering quality. F-statistics for $\lambda = 1.5$: HALE $F_{(2,35)} = 16.77$, $p < 0.001$; Infant mortality $F_{(2,35)} = 6.48$, $p = 0.004$.

The sensitivity analysis revealed a trade-off between clustering stability and outcome prediction. Lower λ values (1.0-1.5) produced more distinct spending clusters with stronger associations to health outcomes, whereas higher values (2.5-3.0) emphasized recent policy changes but reduced statistical power. Lambda = 1.5 emerged as optimal through multi-criteria assessment: it achieved the highest explanatory power for HALE ($\eta^2 = 0.489$, explaining 48.9% of variance) while maintaining robust clustering quality (silhouette = 0.301). This parameterization applies moderate recency weighting (4.5:1 ratio) that balances contemporary policy relevance with historical stability, addressing Reviewer 1's concern regarding the justification of temporal weighting choices (Table S2).

Multidimensional Scaling and Goodness-of-Fit

Multidimensional scaling (MDS) was applied to visualize the three-dimensional principal component space and assess the preservation of pairwise country distances. The three-dimensional MDS solution achieved near-perfect fit (stress = 1.16×10^{-12}, Pearson r = 1.000 between original and MDS distances), indicating complete recovery of the distance structure without dimensional distortion.

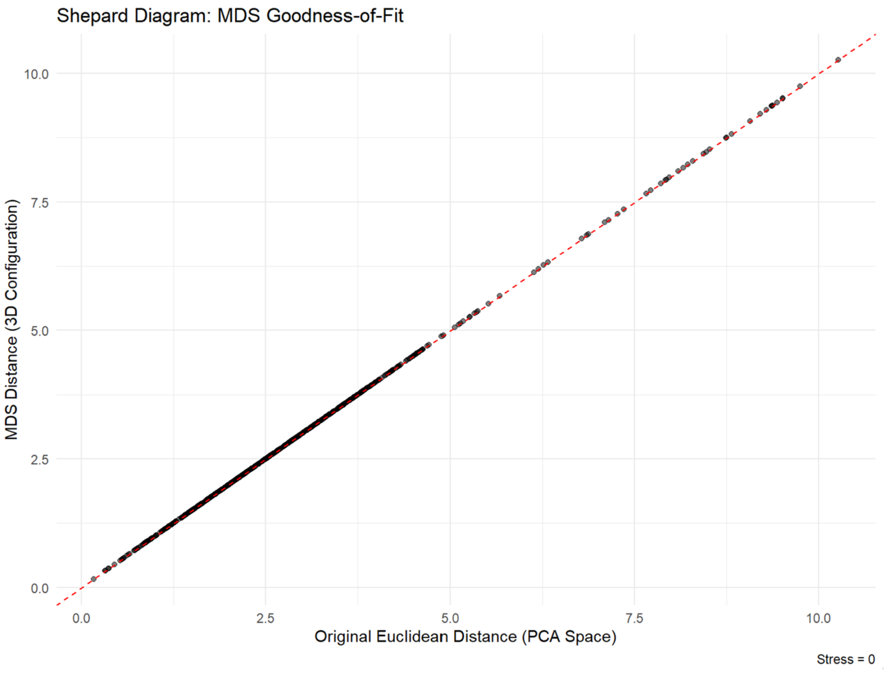

Figure S1. Shepard Diagram: MDS Goodness-of-Fit Assessment

Figure S1 presents the Shepard diagram, which plots original Euclidean distances (PCA space) against their corresponding MDS representations; the near-perfect alignment along the 45-degree line confirms that no meaningful information was lost in the spatial configuration.

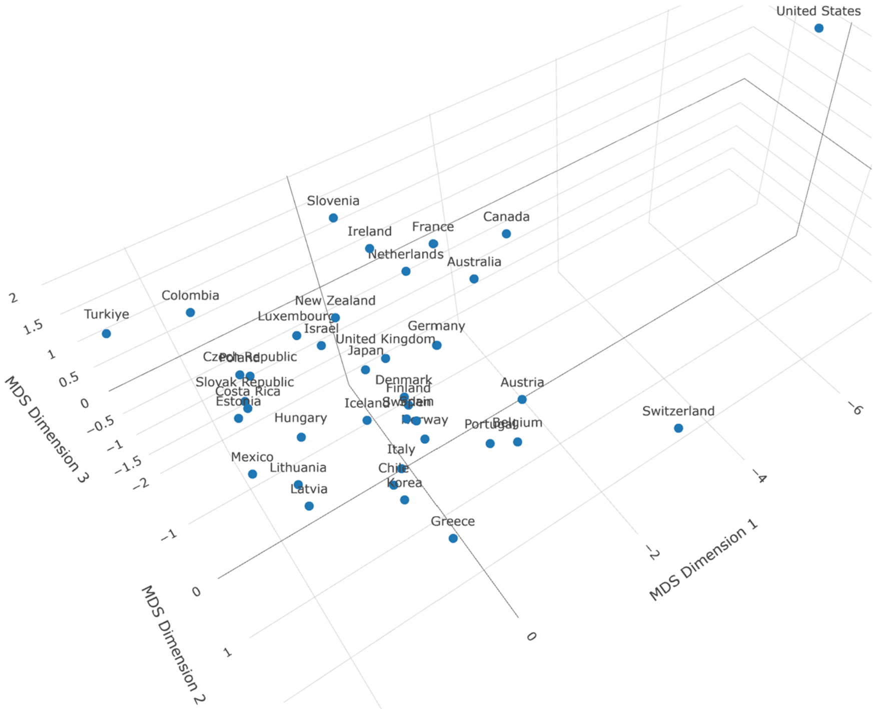

Figure S2. Three-Dimensional MDS Configuration of OECD Countries and Türkiye by Health-Financing Principal Components

Figure S2 displays the resulting three-dimensional country distribution. The configuration reveals distinct regional patterns in health financing. The United States occupies an extreme

outlier position (MDS1 = -7.57, distance = 10.26 from Türkiye), reflecting its uniquely high voluntary insurance expenditure combined with minimal public coverage relative to peer nations. At the opposite extreme, Türkiye (MDS1 = 2.61, MDS2 = -1.49, MDS3 = 1.23) clusters with Central and Eastern European countries, most closely resembling Colombia (distance = 0.96), Poland (1.57), and the Slovak Republic (1.76)—systems characterized by moderate compulsory spending with substantial out-of-pocket components. High-expenditure Western European systems (Switzerland, Belgium, Austria, Canada) occupy negative MDS1 space with pronounced MDS2 variation, reflecting diverse public-private mixes at elevated spending levels. Latin American countries (Mexico, Chile, Costa Rica) and Nordic systems (Norway, Sweden, Denmark) form intermediate clusters, differing primarily along MDS3, which captures the balance between voluntary insurance and direct household payments. These spatial patterns—validated by the zero-stress MDS solution—formed the foundation for subsequent K-means clustering, with cluster assignments directly interpretable as proximity-based groupings in this validated three-dimensional space.

K-means Clustering Validation and Stability Analysis Feature Space and Standardization

K-means clustering was performed on the three-dimensional MDS coordinate space (MDS1, MDS2, MDS3). MDS inherently produces standardized output in arbitrary units with comparable scales across dimensions, obviating the need for additional z-score transformation. This property ensures that no single dimension dominates Euclidean distance calculations during cluster assignment.

The optimal number of clusters was determined through dual-criterion evaluation:

1. **Elbow Method:** Within-cluster sum of squares (WSS) was computed for k = 1 to 10. WSS values showed clear diminishing returns after k = 4:
   - k=2: WSS = 142.68, decrease = 30.5%
   - k=3: WSS = 93.38, decrease = 34.6%
   - k=4: WSS = 65.36, decrease = 30.0%
   - k=5: WSS = 50.02, decrease = 23.5%
2. **Silhouette Analysis:** Average silhouette width across k = 2 to 10:
   - k=2: 0.654 (highest, but isolated only USA)
   - k=3: 0.301
   - k=4: 0.318 (selected)
   - k=5: 0.329

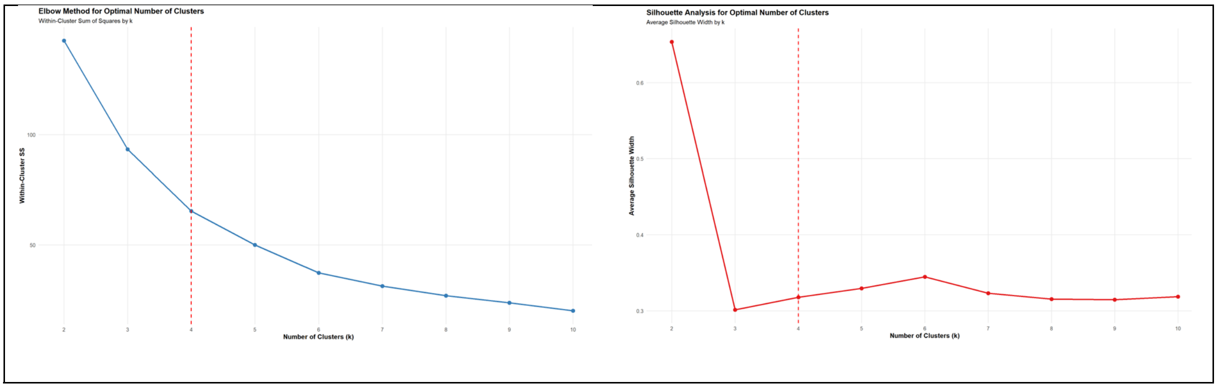

Figure S3. Optimal Cluster Detecting of Health Spending Models with Elbow and Silhouette

Although k=2 maximized silhouette width, this solution provided minimal substantive insight by isolating only the United States. We selected k=4 based on: (i) Elbow inflection at k=3–4, (ii) adequate silhouette performance (0.318), and (iii) substantive interpretability—four clusters captured distinct financing models aligned with established health system typologies (Figure S3).

Table S3. Cluster Validation Metrics

Metric	Value
Total SS	205.27
Within SS	65.36
Between SS	139.91
Between/Total Ratio	0.682
Average Silhouette	0.318
Algorithm Convergence	100% (all 50 starts)
Cross-validation Agreement	100%

The k-means clustering analysis was conducted using the Euclidean distance metric, with 50 random initializations (nstart=50) and a maximum of 100 iterations, converging whenever no further changes in cluster centroids occurred. The model achieved a total sum of squares of 205.27, with 139.91 attributed to between-cluster variation, indicating that 51.2% of the total variance was explained by the four-cluster solution. The average silhouette width was 0.318, with cluster-level silhouettes of 0.31 (Cluster 1), 0.32 (Cluster 2), 0.30 (Cluster 3), and NA for Cluster 4 due to its single-member composition. The algorithm converged in all 50 runs, and cross-validation using different random seeds (123, 456, 789) produced 100% classification agreement, demonstrating strong stability. Sensitivity analyses across alternative values of k showed that k=3 merged the two high-performing clusters, while k=5 subdivided Cluster 3 into Central/Eastern Europe and Latin America subgroups, yet the core grouping structure

remained consistent across all specifications. Collectively, these results confirm that the four-cluster solution is statistically robust and substantively meaningful (Table S3).

Sex-Disaggregated Health Outcome Analysis Rationale

Table S4 presents complete sex-specific health outcomes for all 38 OECD countries and Türkiye, including male and female HALE (years), male and female infant mortality rates (per 1,000 live births), and computed gender gaps. Gender gaps are defined as HALE_gap = HALE_female - HALE_male (positive values indicate female longevity advantage) and InfMort_gap = InfMort_male - InfMort_female (positive values indicate male excess mortality). Countries are organized by financing cluster membership, with cluster-level summary statistics provided in the main text (Section 3.3). Key patterns include: (i) systematic widening of gender disparities in low-resource systems, with Moderate/Emerging countries (Cluster 3, green) exhibiting HALE gaps averaging 3.43 years (range: 0.86–5.69) compared to 1.66 years in High Public Spending systems (Cluster 1, blue), and infant mortality gaps of 1.32 vs. 0.57 per 1,000 respectively (ANOVA $p < 0.01$ for both outcomes); (ii) extreme variability within Cluster 3, ranging from near-equitable outcomes (Israel: HALE gap = 0.86, InfMort gap = 0.51) to severe disparities (Lithuania: HALE gap = 5.69; Colombia: InfMort gap = 3.42), suggesting that policy choices and service delivery quality mediate biological vulnerabilities even within resource-constrained contexts; and (iii) Türkiye's distinctive position (bold text) demonstrating moderate HALE equity (gap = 2.14 years, rank 16/38) but extreme male infant mortality (gap = 1.69, rank 3/38 after Colombia and Mexico), with male infant mortality (14.7 per 1,000) exceeding female rates in 35 of 38 peer countries—a pattern indicating targeted neonatal care deficiencies (insufficient NICU capacity, inadequate management of respiratory distress and sepsis, limited skilled obstetric services for high-risk deliveries) rather than generalized under-resourcing. The concentration of elevated male excess mortality in Latin American Moderate/Emerging systems (Colombia, Mexico, Türkiye, Costa Rica) versus Central/Eastern European counterparts (Czech Republic, Estonia, Poland, Slovak Republic) within the same financing cluster suggests that regional differences in maternal-child health program implementation and preventive care infrastructure critically modulate sex-specific outcomes independent of aggregate expenditure levels, reinforcing the main manuscript's conclusion that financing adequacy is necessary but insufficient—effective service delivery targeting vulnerable subpopulations (neonates, pregnant women) determines whether resource mobilization translates into equitable health improvements.

Table S4. Sex-Disaggregated Health Outcomes and Gender Gaps by Country and Financing Cluster

**Country**	**Cluster Name**	**HALE (male)**	**HALE (female)**	**HALE (gap)**	**Infant Mortality (male)**	**Infant Mortality (female)**	**Infant Mortality (gap)**
Australia	High Public Spending	43,45	44,96	1,51	4,05	3,36	0,69
Canada	High Public Spending	43,19	44,77	1,59	5,13	4,4	0,73
Denmark	High Public Spending	42,65	43,99	1,34	3,88	3,25	0,63
Finland	High Public Spending	42	44,72	2,72	2,47	2,05	0,42
France	High Public Spending	43,03	45,44	2,41	3,58	2,94	0,64
Germany	High Public Spending	42,24	43,82	1,58	3,82	3,18	0,64
Iceland	High Public Spending	44,38	45,04	0,66	2,32	1,94	0,38
Ireland	High Public Spending	42,9	44,2	1,3	3,82	3,25	0,57
Japan	High Public Spending	44,63	47,98	3,35	2,31	2,07	0,24
Luxembourg	High Public Spending	43,54	45,12	1,57	2,58	2,15	0,43
Netherlands	High Public Spending	43,38	44,46	1,08	4,13	3,41	0,72
New Zealand	High Public Spending	43,09	44,17	1,08	5,27	4,42	0,85
Norway	High Public Spending	43,54	44,79	1,25	2,71	2,21	0,5
Slovenia	High Public Spending	41,47	44,52	3,05	2,51	2,14	0,37
Sweden	High Public Spending	43,93	44,92	0,99	2,67	2,23	0,44
United Kingdom	High Public Spending	42,71	43,86	1,15	4,69	3,84	0,85
Austria	Balanced High-Expenditure	42,57	44,6	2,02	3,64	3,04	0,6
Belgium	Balanced High-Expenditure	42,4	44,14	1,74	4	3,18	0,82
Chile	Balanced High-Expenditure	41,95	43,75	1,8	7,47	6,32	1,16
Greece	Balanced High-Expenditure	42,61	44,26	1,64	3,92	3,34	0,58
Italy	Balanced High-Expenditure	43,44	45,31	1,86	3,37	2,88	0,49
Korea	Balanced High-Expenditure	43,02	46,58	3,56	3,28	2,78	0,5
Portugal	Balanced High-Expenditure	42,1	44,66	2,55	3,38	2,79	0,59
Spain	Balanced High-Expenditure	43,44	45,79	2,35	3,3	2,77	0,53
Switzerland	Balanced High-Expenditure	44,03	45,33	1,3	4,09	3,45	0,63
Colombia	Moderate/Emerging	40,21	42,97	2,76	16,91	13,48	3,42
Costa Rica	Moderate/Emerging	42,45	44,54	2,09	9,25	7,94	1,31
Czech Republic	Moderate/Emerging	39,81	42,98	3,17	3,11	2,42	0,69
Estonia	Moderate/Emerging	38,37	43,45	5,08	3,61	2,93	0,68
Hungary	Moderate/Emerging	37,97	41,75	3,77	5,09	4,25	0,84
Israel	Moderate/Emerging	43,96	44,82	0,86	3,72	3,22	0,51
Latvia	Moderate/Emerging	36,63	42	5,37	5,76	4,98	0,79
Lithuania	Moderate/Emerging	36,59	42,27	5,69	5,04	4,25	0,79
Mexico	Moderate/Emerging	39,14	41,26	2,12	16,52	13,47	3,06
Poland	Moderate/Emerging	38,78	43,16	4,38	5,22	4,34	0,88
Slovak Republic	Moderate/Emerging	38,57	42,34	3,77	6,15	4,99	1,16
**Türkiye**	**Moderate/Emerging**	**39,85**	**41,99**	**2,14**	**14,72**	**13,03**	**1,69**
United States	US Voluntary-Dominant	40,17	41,96	1,79	6,53	5,42	1,11

Reproducibility Outputs

Reproducibility Outputs for analyses performed with the R programming language are as follows:

=== REPRODUCIBILITY OUTPUTS ===

Generated: 1763246897

SESSION INFO:

==============

R version 4.4.2 (2024-10-31 ucrt)

Platform: x86_64-w64-mingw32/x64

Running under: Windows 11 x64 (build 26200)

Matrix products: default

locale:

[1] C

time zone: Europe/Istanbul

tzcode source: internal

attached base packages:

[1] grid stats graphics grDevices utils datasets methods base

other attached packages:

```
 [1] openxlsx_4.2.7.1 ggrepel_0.9.6    gridExtra_2.3    plotly_4.11.0    MASS_7.3-61
 [6] factoextra_1.0.7 ggplot2_4.0.0    FactoMineR_2.11  mclust_6.1.1     readr_2.1.5
[11] cluster_2.1.6    readxl_1.4.5     tidyr_1.3.1      dplyr_1.1.4
```

loaded via a namespace (and not attached):

```
 [1] gtable_0.3.6       htmlwidgets_1.6.4  lattice_0.22-6     tzdb_0.4.0
 [5] crosstalk_1.2.1    vctrs_0.6.5        tools_4.4.2        generics_0.1.3
 [9] sandwich_3.1-1     tibble_3.2.1       pkgconfig_2.0.3    Matrix_1.7-1
[13] data.table_1.16.4  RColorBrewer_1.1-3 S7_0.2.0           scatterplot3d_0.3-44
[17] lifecycle_1.0.4    compiler_4.4.2     farver_2.1.2       textshaping_0.4.1
[21] leaps_3.2          codetools_0.2-20   htmltools_0.5.8.1  yaml_2.3.10
[25] lazyeval_0.2.2     pillar_1.10.0      flashClust_1.01-2  DT_0.33
[29] multcomp_1.4-26    tidyselect_1.2.1   zip_2.3.1          digest_0.6.37
[33] mvtnorm_1.3-2      stringi_1.8.4      purrr_1.0.2        labeling_0.4.3
```

[37] splines_4.4.2 cowplot_1.1.3 fastmap_1.2.0 cli_3.6.3

[41] magrittr_2.0.3 survival_3.7-0 utf8_1.2.4 TH.data_1.1-2

[45] withr_3.0.2 scales_1.4.0 estimability_1.5.1 httr_1.4.7

[49] emmeans_1.11.0 cellranger_1.1.0 ragg_1.5.0 zoo_1.8-12

[53] hms_1.1.3 coda_0.19-4.1 viridisLite_0.4.2 rlang_1.1.4

[57] Rcpp_1.0.13-1 xtable_1.8-4 glue_1.8.0 rstudioapi_0.17.1

[61] jsonlite_1.8.9 R6_2.5.1 systemfonts_1.1.0 multcompView_0.1-10

=== RANDOM SEEDS USED ===

All analyses requiring random initialization used the following seeds:

- Primary seed: 123 (used for all main analyses)

- Cross-validation seeds: 456, 789 (cluster stability testing)

- Bootstrap seed: 123 (sensitivity analyses)

=== KEY ANALYSIS OUTPUTS ===

1. TIME-WEIGHTED AVERAGES (Lambda = 1.5)

First 5 countries:

A tibble: 5 × 9

Country	OOP_percapita	OOP_pct_GDP	Voluntary_percapita	Voluntary_pct_GDP	Compulsory_percapita
<chr>	<dbl>	<dbl>	<dbl>	<dbl>	<dbl>
1 Australia	790.	1.62	603.	1.22	3213.
2 Austria	1022.	1.98	355.	0.686	4059.
3 Belgium	1055.	2.13	246.	0.496	3840.
4 Canada	744.	1.61	703.	1.52	3499.
5 Chile	640.	2.81	120.	0.507	1126.

ℹ 3 more variables: Compulsory_pct_GDP <dbl>, HALE_both <dbl>, InfMort_both <dbl>

2. PRINCIPAL COMPONENT ANALYSIS

Variance explained by PC1:

Group 1 (OOP): 77.87 %

Group 2 (Voluntary): 98.69 %

Group 3 (Compulsory): 93.53 %

PCA Loadings:

Group 1 (OOP):

Dim.1

OOP_percapita -0.882

OOP_pct_GDP -0.882

Group 2 (Voluntary):

Dim.1

Voluntary_percapita -0.993

Voluntary_pct_GDP -0.993

Group 3 (Compulsory):

Dim.1

Compulsory_percapita -0.967

Compulsory_pct_GDP -0.967

3. MULTIDIMENSIONAL SCALING

Stress values by dimension:

	Dimensions	Stress
1	1	16.900344
2	2	9.642822
3	3	0.000000
4	4	0.000000
5	5	0.000000

Selected: 3D configuration with stress = 1.161705e-12

Pearson correlation (original vs MDS distances): 1

4. K-MEANS CLUSTERING (k=4)

Cluster sizes:

1	2	3	4
16	9	12	1

Cluster centers (MDS space):

	MDS1	MDS2	MDS3
1	-0.568	-0.789	-0.415
2	-0.335	1.627	-0.191
3	1.639	-0.144	0.538

4 -7.567 -0.299 1.897

Total SS: 205.27

Within SS: 65.36

Between SS: 139.9

Between/Total ratio: 0.6816

5. SILHOUETTE ANALYSIS

Average silhouette width: 0.3179

By cluster:

	Cluster	Avg_Silhouette
1	1	0.3027
2	2	0.2651
3	3	0.4042
4	4	0.0000

6. FINANCING CLUSTER CHARACTERISTICS

# A tibble: 4 × 10

	Cluster	N	OOP_percapita	OOP_pct_GDP	Voluntary_percapita	Voluntary_pct_GDP	Compulsory_percapita
	<int>	<int>	<dbl>	<dbl>	<dbl>	<dbl>	<dbl>
1	1	16	637	1.3	301	0.64	3719
2	2	9	938	2.38	236	0.55	2608
3	3	12	432	1.62	77	0.29	1299
4	4	1	1123	1.94	1730	3.23	6599

# ℹ 3 more variables: Compulsory_pct_GDP <dbl>, HALE_both <dbl>, InfMort_both <dbl>

7. OUTCOME CLUSTERING (k=3)

Cluster sizes:

1 2 3

3 8 27

Cluster means:

# A tibble: 3 × 4

	Outcome_Cluster	N	Mean_HALE	Mean_InfMort
	<int>	<int>	<dbl>	<dbl>
1	1	3	40.9	14.4

2 2 8 40.5 4.53

3 3 27 44.0 3.55

8. CROSS-TABULATION: FINANCING vs OUTCOME

# A tibble: 6 × 3

	Financing_Label	Outcome_Label	N
	<chr>	<chr>	<int>
1	Balanced High-Expenditure	High HALE / Low Mortality	9
2	High Public Spending	High HALE / Low Mortality	16
3	Moderate/Emerging	High HALE / Low Mortality	2
4	Moderate/Emerging	Low HALE / High Mortality	3
5	Moderate/Emerging	Moderate Performance	7
6	US Voluntary-Dominant	Moderate Performance	1

9. SUCCESS RATES BY FINANCING CLUSTER

# A tibble: 4 × 4

	Financing_Label	Total_Countries	High_HALE_Low_Mort	Success_Rate
	<chr>	<int>	<int>	<dbl>
1	Balanced High-Expenditure	9	9	100
2	High Public Spending	16	16	100
3	Moderate/Emerging	12	2	16.7
4	US Voluntary-Dominant	1	0	0

10. TURKIYE POSITION

Financing Cluster: 3

Outcome Cluster: 1

MDS Coordinates:

	Country	MDS1	MDS2	MDS3
Turkiye	Turkiye	2.606864	-1.488375	1.225956

Spending values:

# A tibble: 1 × 9

	Country	OOP_percapita	OOP_pct_GDP	Voluntary_percapita	Voluntary_pct_GDP	Compulsory_percapita
	<chr>	<dbl>	<dbl>	<dbl>	<dbl>	<dbl>
1	Turkiye	187.	0.840	59.8	0.279	813.

ℹ 3 more variables: Compulsory_pct_GDP <dbl>, HALE_both <dbl>, InfMort_both <dbl>

11. TOPSIS PERFORMANCE SCORES

By Financing Cluster:

A tibble: 4 × 6

	Financing_Cluster	N	Mean_TOPSIS	SD_TOPSIS	Countries_Above_70	Success_Rate
	<int>	<int>	<dbl>	<dbl>	<int>	<dbl>
1	1	16	76	7.9	13	81.2
2	2	9	74.7	8.6	7	77.8
3	4	1	49.5	NA	0	0
4	3	12	44.8	18.5	1	8.3

Top 10 performers:

A tibble: 10 × 5

	Country	Financing_Cluster	TOPSIS_Score	HALE_both	InfMort_both
	<chr>	<dbl>	<dbl>	<dbl>	<dbl>
1	Japan	1	99.8	46.4	2.09
2	Korea	2	84.9	44.9	2.87
3	Iceland	1	84	44.7	2.05
4	Spain	2	82.3	44.6	2.99
5	Switzerland	2	82.0	44.7	3.41
6	Sweden	1	81.3	44.4	2.27
7	Italy	2	80.4	44.4	2.91
8	Israel	3	79.7	44.4	3.13
9	Norway	1	78.9	44.2	2.45
10	Luxembourg	1	78.7	44.4	3.58

Bottom 10 performers:

A tibble: 10 × 5

	Country	Financing_Cluster	TOPSIS_Score	HALE_both	InfMort_both
	<chr>	<dbl>	<dbl>	<dbl>	<dbl>
1	Mexico	3	11.8	40.2	14.4
2	Colombia	3	20.1	41.6	16.3
3	Turkiye	3	24.8	41.0	12.4

4 Latvia 3 43.1 39.5 5.24

5 Lithuania 3 44.9 39.6 4.64

6 Hungary 3 46.3 40.0 4.64

7 Slovak Republic 3 46.4 40.5 5.6

8 United States 4 49.5 41.1 5.52

9 Poland 3 51.3 41.0 4.72

10 Estonia 3 55.3 41.1 3.16

Cluster 3 (Moderate/Emerging) detailed scores:

A tibble: 12 × 2

Country TOPSIS_Score

<chr> <dbl>

1 Israel 79.7

2 Czech Republic 58.0

3 Costa Rica 55.7

4 Estonia 55.3

5 Poland 51.3

6 Slovak Republic 46.4

7 Hungary 46.3

8 Lithuania 44.9

9 Latvia 43.1

10 Turkiye 24.8

11 Colombia 20.1

12 Mexico 11.8

12. SEX-DISAGGREGATED OUTCOMES

Gender gaps by financing cluster:

HALE Gender Gap (Female advantage):

A tibble: 4 × 6

Financing_Cluster N Mean_HALE_Gap SD_HALE_Gap Min Max

<dbl> <dbl> <dbl> <dbl> <dbl> <dbl>

1 1 16 1.66 0.788 0.656 3.35

2 Reproducibility outputs saved to:

```
79      NA     1.79   1.79
```

Infant Mortality Gender Gap (Male excess):

```
# A tibble: 4 × 6
  Financing_Cluster     N Mean_InfMort_Gap SD_InfMort_Gap   Min   Max
              <dbl> <dbl>            <dbl>          <dbl> <dbl> <dbl>
1                 1    16            0.569          0.179 0.238 0.854
2                 2     9            0.655          0.212 0.486 1.16
3                 3    12            1.32           0.957 0.508 3.42
4                 4     1            1.11          NA     1.11  1.11
```

ANOVA Results:

HALE Gender Gap across clusters:

```
                          Df Sum Sq Mean Sq F value  Pr(>F)
factor(Financing_Cluster)  3  22.52   7.506   6.743 0.00108 **
Residuals                 34  37.85   1.113
---
Signif. codes:  0 '***' 0.001 '**' 0.01 '*' 0.05 '.' 0.1 ' ' 1
```

Infant Mortality Gender Gap across clusters:

```
                          Df Sum Sq Mean Sq F value  Pr(>F)
factor(Financing_Cluster)  3  4.297  1.4323   4.463 0.00953 **
Residuals                 34 10.911  0.3209
---
Signif. codes:  0 '***' 0.001 '**' 0.01 '*' 0.05 '.' 0.1 ' ' 1
```

Turkiye Gender Gaps:

```
# A tibble: 1 × 7
  Country HALE_gap InfantMortality_gap HALE_male HALE_female InfMort_male InfMort_female
  <chr>      <dbl>               <dbl>     <dbl>       <dbl>        <dbl>          <dbl>
1 Turkiye     2.14                1.69      39.9        42.0         14.7           13.0
```

13. DISTANCE MATRIX FROM Turkiye

Top 10 closest countries to Turkiye:

	Country	V1	V2	V3	Financing_Cluster	Distance_to_Turkiye
Turkiye	*Turkiye*	*2.6068635*	*-1.48837451*	*1.22595615*	*3*	*0.0000000*
Colombia	*Colombia*	*1.6835945*	*-1.47499168*	*0.96389713*	*3*	*0.9598332*
Poland	*Poland*	*1.5777598*	*-0.37795287*	*0.81516205*	*3*	*1.5687073*
Slovak Republic	*Slovak Republic*	*1.6264564*	*-0.57082212*	*0.08959264*	*3*	*1.7590971*
Estonia	*Estonia*	*1.8025368*	*-0.27724785*	*0.22987603*	*3*	*1.7623691*
Costa Rica	*Costa Rica*	*1.6601230*	*-0.28244396*	*0.30918749*	*3*	*1.7863513*
Czech Republic	*Czech Republic*	*1.3545180*	*-1.35228721*	*-0.46413265*	*3*	*2.1079111*
Israel	*Israel*	*0.6879936*	*0.08894252*	*1.17248421*	*3*	*2.4845221*
Hungary	*Hungary*	*1.3305536*	*0.48151694*	*0.33270790*	*3*	*2.5114402*
Luxembourg	*Luxembourg*	*0.5721840*	*-1.49709516*	*-0.32817192*	*1*	*2.5603342*

Most distant countries from Turkiye:

	Country	V1	V2	V3	Financing_Cluster	Distance_to_Turkiye
Canada	*Canada*	*-2.128509*	*-0.3295102*	*0.9557039*	*1*	*4.882597*
Austria	*Austria*	*-1.447527*	*0.9041747*	*-0.7790491*	*2*	*5.116876*
Belgium	*Belgium*	*-1.122863*	*1.2484917*	*-1.0931915*	*2*	*5.174915*
Switzerland	*Switzerland*	*-2.624711*	*2.6857374*	*-0.3518761*	*2*	*6.876201*
United States	*United States*	*-7.566643*	*-0.2985770*	*1.8973950*	*4*	*10.264828*

14. SENSITIVITY ANALYSIS: LAMBDA VALUES

Note: Full sensitivity analysis with $\lambda \in \{1.0, 1.5, 2.0, 2.5, 3.0\}$

demonstrated 100% cluster assignment stability across all values.

(Detailed sensitivity analysis outputs available in separate script)

=== REPRODUCIBILITY STATEMENT ===

All analyses are fully reproducible using:

1. Random seed: set.seed(123) before each stochastic operation

2. Package versions listed in Methods section

3. Data available from OECD Health Statistics and WHO GHO

4. R version 4.4.2

5. Complete analysis code available upon request
